# Manipulation of Host Cholesterol by SARS-CoV-2

**DOI:** 10.1101/2024.11.13.623299

**Published:** 2024-11-14

**Authors:** Aliza Doyle, Baley A. Goodson, Oralia M. Kolaczkowski, Rui Liu, Jingyue Jia, Hu Wang, Xianlin Han, Chunyan Ye, Steven B. Bradfute, Alison M. Kell, Monica Rosas Lemus, Jing Pu

**Affiliations:** 1 Department of Molecular Genetics and Microbiology, University of New Mexico Health Sciences Center, Albuquerque, New Mexico 87131, USA; 2 Department of Pharmaceutical Sciences, College of Pharmacy, University of New Mexico Health Sciences Center, Albuquerque, New Mexico 87131, USA; 3 Department of Internal Medicine, University of New Mexico Health Sciences Center, Albuquerque, New Mexico 87131, USA; 4 Autophagy, Inflammation, and Metabolism Center of Biomedical Research Excellence, University of New Mexico Health Sciences Center, Albuquerque, New Mexico 87131, USA; 5 Department of Medicine, UT Health San Antonio Long School of Medicine, San Antonio, Texas 78229, USA

**Keywords:** Cellular cholesterol transport, bis(monoacylglycero)phosphate (BMP), lipidomics, virus-host interaction

## Abstract

SARS-CoV-2 infection is associated with alterations in host lipid metabolism, including disruptions in cholesterol homeostasis. However, the specific mechanisms by which viral proteins influence cholesterol remain incompletely understood. Here, we report that SARS-CoV-2 infection induces cholesterol sequestration within lysosomes, with the viral protein ORF3a identified as the primary driver of this effect. Mechanistically, we found that ORF3a interacts directly with the HOPS complex subunit VPS39 through a hydrophobic interface formed by residues W193 and Y184. A W193A mutation in ORF3a significantly rescues cholesterol egress and corrects the mislocalization of the lysosomal cholesterol transporter NPC2, which is caused by defective trafficking of the trans-Golgi network (TGN) sorting receptor, the cation-independent mannose-6-phosphate receptor (CI-MPR). We further observed a marked reduction in bis(monoacylglycero)phosphate (BMP), a lipid essential for lysosomal cholesterol egress, in both SARS-CoV-2-infected cells and ORF3a-expressing cells, suggesting BMP reduction as an additional mechanism of SARS-CoV-2-caused cholesterol sequestration. Inhibition of lysosomal cholesterol egress using the compound U18666A significantly decreased SARS-CoV-2 infection, highlighting a potential viral strategy of manipulating lysosomal cholesterol to modulate host cell susceptibility. Our findings reveal that SARS-CoV-2 ORF3a disrupts cellular cholesterol transport by altering lysosomal protein trafficking and BMP levels, providing new insights into virus-host interactions that contribute to lipid dysregulation in infected cells.

## INTRODUCTION

COVID-19, caused by the SARS-CoV-2 virus, has not only led to respiratory and systemic complications but has also been associated with significant disruptions in lipid metabolism, e.g., cholesterol homeostasis ^1–5^. Emerging evidence suggests that abnormal cholesterol levels persist not only during infection but also in the post-acute infection phases of the disease. COVID-19 patients frequently exhibit reduced serum cholesterol levels, which correlate with disease severity^1,2^, reflecting the virus’s impact on metabolic processes. In cases of long COVID, there is evidence of an increased risk of dyslipidemia and cardiovascular diseases, marked by elevated low-density lipoprotein (LDL) cholesterol and reduced high-density lipoprotein (HDL) cholesterol levels ^4,5^. Understanding the mechanisms driving these metabolic changes is essential to developing therapeutic strategies that target lipid metabolism, with the aim of alleviating COVID-19 severity and reducing the long-term metabolic burdens associated with the disease.

Cholesterol plays a vital role in maintaining cellular structure and function. Thus, cells tightly regulate cholesterol levels through de novo synthesis and uptake mechanisms^6^. Cholesterol uptake primarily involves the uptake of LDL particles from the bloodstream, in which LDL particles are internalized via endocytosis and transported to the cellular degradation organelles, lysosomes, for enzymatic degradation and free cholesterol releasing^6^. The lysosome then serves as a central hub for cholesterol sorting and distribution, ensuring that cholesterol is transported to other cellular compartments to meet the cell’s structural and metabolic demands^7,8^. For examples, the cholesterol arriving at the endoplasmic reticulum serves as a signal to regulate the expression of genes that control cholesterol synthesis and uptake^9^, and the cholesterol arriving at the plasma membrane is the most important donor to the local cholesterol pool that modulate plasma membrane fluidity and stability^10^. SARS-CoV-2 appears to exploit the plasma membrane cholesterol to enhance infectivity^11^, evident by a decreased infection rate after extracting plasma membrane cholesterol^12^. Therefore, the lysosomal cholesterol pathway is critical for cellular cholesterol homeostasis and may play a role in SARS-CoV-2 infection.

Lysosomal cholesterol egress is the first step of cholesterol distribution. Key proteins involved in this pathway include the transmembrane cholesterol transporter Niemann-Pick C1 (NPC1) and the lumenal transporter Niemann-Pick C2 (NPC2), which work together to transport cholesterol out of the lysosome^8^. In addition, other lysosomal proteins, such as lumenal saps and membrane proteins LAMP2^13–15^, facilitate cholesterol egress^8^. The trafficking and localization of newly synthesized lysosome transmembrane proteins requires their sorting signals and clathrin coats, while the lysosome lumenal proteins are mediated by sorting receptors, such as the mannose-6-phosphate receptors (M6PR)^16^. These receptors deliver lumenal proteins from the trans-Golgi network (TGN) to the lysosome and are recycled back to the TGN. Late endosome and lysosome proteins, such as HOPS complex, and TGN proteins, such as GARP complex, facilitate recycle of the sorting receptors^17–19^. Disruption of these transport pathways leads to the failure of targeting newly synthesized proteins to the lysosome and accumulation of cholesterol within lysosomes^19–21^, a hallmark of Niemann-Pick disease type C, caused by genetic mutations in NPC1 or NPC2^22–24^. This lysosomal cholesterol sequestration has profound cellular consequences, disrupting lipid homeostasis and impairing cellular functions dependent on cholesterol, such as membrane dynamics and signaling pathways. Some types of viruses take advantage of the machinery of lysosomal cholesterol transport to fulfil their life cycles, such as that NPC1 is hijacked by Ebola virus for their infection^25^.

Another type of molecules that facilitate lysosomal cholesterol egress is Bis(monoacylglycero)phosphate (BMP, also called lysobisphosphatidic acid or LBPA). BMP is a unique phospholipid enriched in the late endosome and lysosome membrane and plays an important role in sorting and degradation functions of endosomes and lysosomes^9,26–28^. In addition, BMP physically interacts with NPC2 and allows cholesterol to exit the lysosome in an NPC1-independent way, as shown that addition of BMP to NPC1-defecient cells rescues cholesterol sequestration, but it does not affect NPC2-defecient cells^29,30^. In addition to its role in cholesterol transport, BMP has also been implicated in viral infection processes by promoting viral fusion with late endosome membranes^31–35^.

In this study, we report that SARS-CoV-2 infection leads to cholesterol sequestration in lysosomes, primarily driven by the viral protein ORF3a. ORF3a disrupts lysosomal lumenal protein trafficking, including the transport of NPC2, and reduces BMP levels. These defects were primely due to the interaction between ORF3a and the HOPS subunit VPS39. An ORF3a mutant that dampers the interaction mostly rescued the defects. Using a compound to mimic this lysosomal cholesterol egress defect significantly reduced SARS-CoV-2 infectivity, suggesting that SARS-CoV-2 manipulation of host cholesterol may serve as a mechanism to enhance viral survival by reducing secondary infections.

## RESULTS

### Assessment of cellular cholesterol in SARS-CoV-2-infected cells

**Figure 1:**
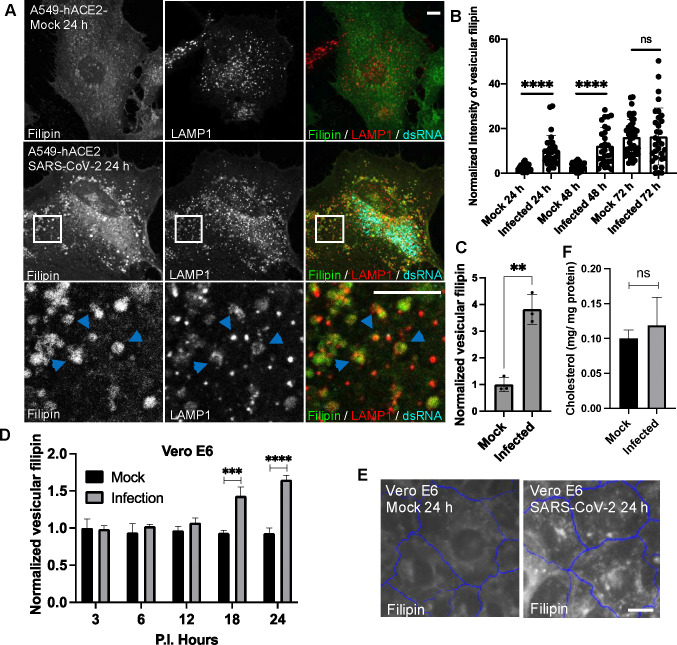
Characterization of cellular free cholesterol in SARS-CoV-2 infected cells **A-C.** A549 cells stably expressing human ACE2 receptor (A549-hACE2) were infected with SARS-CoV-2 and fixed at the indicated time post-infection (P.I.). Filipin, a dsRNA antibody, and a LAMP1 antibody were applied to visualize free cholesterol, identify infected cells, and label lysosomes. Confocal microscopy was performed to collect fluorescence images, and the images at 24 h P.I. were shown in **A,** with arrows pointing colocalized filipin and LAMP1. The intensity of Filipin puncta was quantified with FIJI, and the mean values of total filipin in each cell from three independent experiments were plotted, shown as in **B.** Vesicular filipin was quantified at 24 h P.I. from at least 1,000 infected cells per well and three replicate wells in an experiment by CellInsight microscope platform. Three independent experiments were performed, and pooled results were shown in **C. D,E.** Similar experiments were perform as above in Vero E6 cells, and filipin was quantified at the indicated time points. **F.** Total lipids were extracted from mock and infected A549-hACE2 cells at 24 h P.I. and subjected to free cholesterol measurement with GC-MS. Total protein levels were measure by BCA assays. Two independent experiments with 3 replications were performed. Bar graphs are presented as min-max (B) or mean ± SD (others). *p* values were determined using *t’* test or One-way ANOVA test. **, *p*<0.001, ***, *p*<0.001, ****, *p*<0.0001, n.s., not significant. Scale bars, 5 *μ*m.

To examine how SARS-CoV-2 infection alters host cholesterol at cellular levels, we infected human lung carcinoma epithelial cells A549, stably expressing the viral entry receptor ACE2 of human species (A549-hACE2), with the Washington strain of SARS-CoV-2. We confirmed infection in approximately 80% of cells by immunostaining for double-stranded RNA (dsRNA) (Figs. 1A & S1A). Using filipin staining to detect free cholesterol, we observed a marked increase in filipin signal in infected cells, appearing as punctate structures, compared to mock-treated cells (Fig. 1A). Similar observations were made in a different ACE2-overexpressing A549 cell line (Fig. S1B). Co-staining with an antibody against late endosomal and lysosomal membrane protein LAMP1 revealed that the filipin puncta colocalized with LAMP1-positive vesicles (Fig. 1A), indicating that cholesterol was sequestrated in the late endosomes and lysosomes. Due to the similarity of these two organelles, we refer them to lysosomes hereafter. Quantification of confocal images using FIJI showed that lysosomal cholesterol levels significantly increased at 24- and 48-hours post-infection (Fig. 1B). Although filipin puncta were also present at 72 hours post-infection, mock cells showed elevated filipin signal, likely due to cell responses associated with high cell density^36^ (Fig. 1B). Further analysis of over 1,500 infected cells using a high-content imaging system confirmed a consistent increase in filipin-positive puncta (Fig. 1C). To validate this cholesterol alteration, we infected monkey kidney cells Vero E6 with SARS-CoV-2 and analyzed cholesterol levels over time. High-content imaging showed a slight increase in cholesterol levels at 6 and 12 hours, which became pronounced at 18- and 24-hours post-infection (Figs. 1D, E), confirming that SARS-CoV-2 infection induces an increase in intracellular free cholesterol. This change persisted beyond the viral eclipse phase (~10 hours)^37^. Gas chromatography-mass spectrometry analysis of total cholesterol levels in mock and infected A549 cells showed no significant difference (Fig. 1F), suggesting that SARS-CoV-2 infection mainly alters cholesterol distribution within cells, possibly due to impaired lysosomal cholesterol transport.

### Identification of the viral proteins responsible for increased lysosomal cholesterol

**Figure 2:**
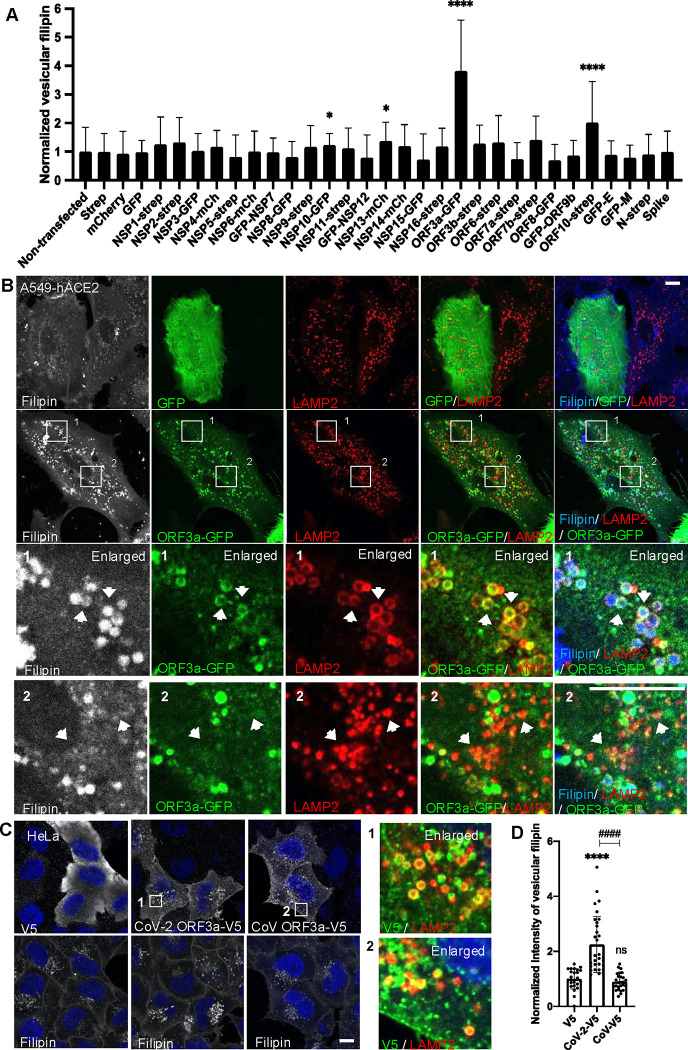
Identification of SARS-CoV-2 proteins responsible for lysosome cholesterol sequestration **A.** Plasmids encoding SARS-CoV-2 proteins were transfected into A549-hACE2 cells individually. Cells were fixed at 24 h post-transfection and co-stained with filipin to visualized free cholesterol and antibodies against the epitope tags to identify transfected cells. Spike protein has no tag and was identified with an antibody. Confocal imaging was performed to randomly take images, and filipin intensity was quantified with FIJI in twenty to fifty cells from two independent experiments. **B.** A549-hACE2 cells were transfected with ORF3a-GFP plasmid as above and co-stained with filipin and GFP and LAMP2 antibodies. **C,D.** HeLa cells were transfected with plasmids encode V5 fused ORF3a proteins in SARS-CoV or SARS-CoV-2. V5 alone served as a control. Staining and quantification was performed as described above. Bar graphs are presented as mean ± SD. *p* values were determined using *t’* test or One-way ANOVA test. *, *p*<0.05, **** (vs. control) or #### (CoV vs CoV-2), *p*<0.0001, n.s., not significant. Scale bars, 5 *μ*m. Framed images in C were enlarged 9.36 folds.

To identify the specific viral proteins responsible for lysosomal cholesterol sequestration, we individually transfected A549-hACE2 cells with 28 plasmids encoding SARS-CoV-2 proteins and quantified filipin puncta 24 hours post-transfection. Most viral proteins (24 out of 28) did not show a significant effect on cholesterol distribution. However, NSP10 and NSP13 had modest effects, ORF10 doubled the filipin signal, and ORF3a notably shows the strongest increase in filipin puncta (Fig. 2A). Unlike NSP10, NSP13, or ORF10, which were mainly cytoplasmic (Fig. S2), ORF3a displayed punctate or halo-like structures that partially co-localized with LAMP2 (Fig. 2A), indicating ORF3a’s association with lysosomal membranes, in line with previous findings^38–40^. Remarkably, ORF3a-positive lysosomes appeared swollen and were filled with filipin (Fig. 1B, arrow in enlarged frame 1), in contrast to ORF3a-negative lysosomes in the same cells, which remained small and displayed minimal filipin signal (Fig. 1B, arrow in enlarged frame 2). This suggests that ORF3a, particularly when localized on lysosomes, plays a critical role in cholesterol sequestration.

To further validate this observation, we transfected HeLa cells with an ORF3a plasmid tagged with a V5 epitope. ORF3a-V5 also formed punctate or halo structures, partially co-localized with LAMP2, and significantly increased filipin signal (Fig. 2C,D), demonstrating that ORF3a enhances lysosomal cholesterol accumulation across different cell types. Additionally, we included ORF3a from SARS-CoV, which shares over 70% sequence identity with SARS-CoV-2 ORF3a, as a comparison. Interestingly, while SARS-CoV ORF3a also displayed a punctate distribution, it did not increase filipin, suggesting a specificity in cholesterol sequestration by SARS-CoV-2 ORF3a (Figs. 2C,D).

Together, these results demonstrate that SARS-CoV-2 infection sequesters host lysosomal cholesterol primarily through the action of ORF3a. Expression of ORF3a alone is sufficient to drive cholesterol accumulation in lysosomes, likely due to its lysosomal localization. The differential effects on lysosomal cholesterol between ORF3a proteins from SARS-CoV and SARS-CoV-2 may offer insights into the underlying mechanisms.

### The Structural difference between SARS-CoV and SARS-CoV-2 ORF3a

**Figure 3:**
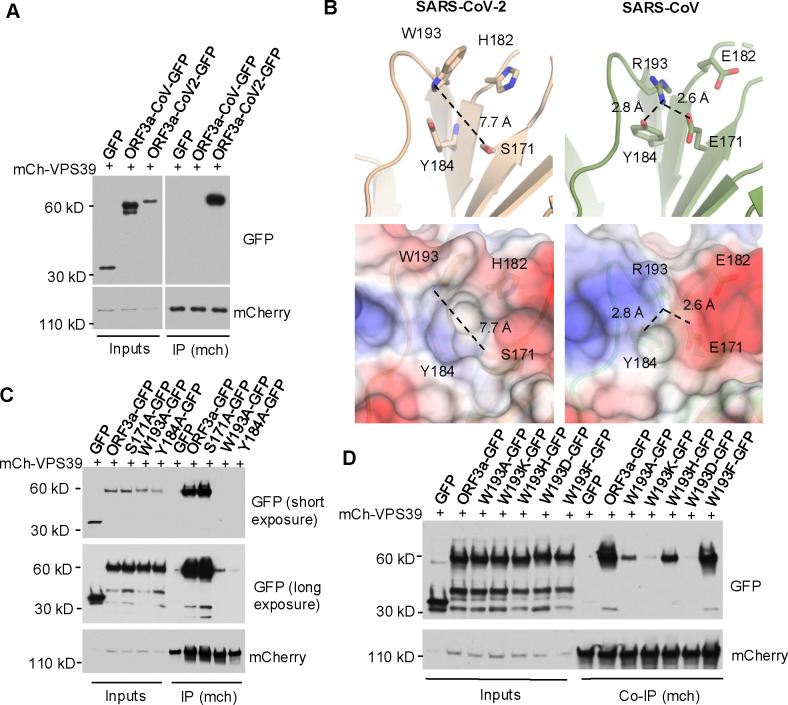
Structural analysis of ORF3a-VPS39 interaction interface in ORF3a **A.** HeLa cells were co-transfected with mCh-VPS39 and different GFP-ORF3a constructs as indicated and lysed in a non-denature detergent-contained buffer for co-immunoprecipitation of mCh-VPS39. Immunoblotting was performed to detect GFP signals. **B.** The structures of ORF3a proteins from SARS-CoV-2 (beige) and SARS-CoV (green) were shown with the cartoon representation of the indicated residues (upper panel). The side chains are depicted as sticks with color code for carbon (beige or green), nitrogen (blue), and oxygen (red). The presence of W193 in SARS-CoV-2 induces an open conformation of the divergent loop that is 7.7 Å away from S171 (dotted line), while R193 in SARS-CoV forms hydrogen bonds with D171 or Y184 (dotted lines). The lower panels represent overlays between electrostatics surface and the cartoon representations. Positively charged surface is depicted in blue, hydrophobic surface in white, and negatively charged surface in red. All images and measurements were performed using PyMol V 2.4.0. **C,D.** Transfection and co-immunoprecipitation was performed as described in A to detect the interaction between mCh-VPS39 and GFP-tagged ORF3a and its mutants.

Previous studies revealed that SARS-CoV-2 ORF3a, but not SARS-CoV ORF3a, interacts with the host protein VPS39^38–41^ (Figs. 3A & S3B), a subunit of the HOPS complex. SARS-CoV-2 infection did not downregulate VPS39 or another HOPS subunit VPS11 expression levels (Fig. S3A). Notably, our recent work showed that the HOPS complex is essential for lysosomal cholesterol egress^19^. Based on these findings, we hypothesized that SARS-CoV-2 ORF3a disrupts lysosomal cholesterol egress by inhibiting VPS39. This hypothesis aligns with the observation that SARS-CoV ORF3a does not interact with VPS39^39^ (Fig. 3A) and did not alter lysosomal cholesterol (Figs. 2C,D). To test this, we aimed to disrupt the ORF3a-VPS39 interaction by introducing specific point mutations.

Miao et al. identified the residues S171 and W193 in SARS-CoV-2 ORF3a as crucial for its interaction with VPS39, as mutating these residues to those in SARS-CoV ORF3a abolished the interaction^39^. Structural analysis of SARS-CoV-2 ORF3a^42,43^ revealed that W193, Y184, S171, and H182 cluster near each other (Fig. 3B, upper), with W193 and Y184 forming a hydrophobic surface likely involved in protein-protein interactions, while S171 and H182 are positioned outside this hydrophobic surface (Fig. 3B, lower). In our alanine mutation screen, we found that W193A and Y184A mutations weakened or disrupted the interaction, whereas S171A or H182A mutations did not (Figs. 3C & S3C,D). This indicates that W193 and Y184, but not S171 or H182, are essential for binding. Further structural analysis indicates that the hydrophobic surface formed by W193 and Y184 (7.7 Å) in SARS-CoV-2 ORF3a is occluded in SARS-CoV ORF3a due to a hydrogen bond between R193 and E171 (2.6 Å) and Y184 (2.8 Å), resulting a “closed conformation” (Fig. 3B, upper). This model is supported by the observation that the S171E mutation impairs the interaction^39^, likely due to the large hydrophilic side chain of glutamic acid obstructing the hydrophobic surface formed by W193 and Y184, thereby blocking the interaction. Collectively, these findings suggest that W193 and Y184 form the primary binding site, while S171 and H182 contribute to an optimal binding environment.

Given that both W193 and Y184 are aromatic amino acids, we hypothesized that their aromatic rings play a role in the interaction. To test this, we mutated W193 in SARS-CoV-2 ORF3a to various amino acids and evaluated the effects on binding. Replacing W193 with another hydrophobic aromatic amino acid, such as phenylalanine, preserved the interaction. However, substituting W193 with a charged aromatic amino acid, like histidine, or with a hydrophobic but non-aromatic amino acid, like alanine, reduced binding. Furthermore, replacing W193 with a charged, non-aromatic amino acid, such as lysine or aspartic acid, nearly abolished the interaction (Fig. 3F). These results suggest that both the aromatic ring and hydrophobicity of W193 are critical for the binding of SARS-CoV-2 ORF3a to VPS39.

### ORF3a-VPS39 interaction interrupts NPC2 trafficking

**Figure 4:**
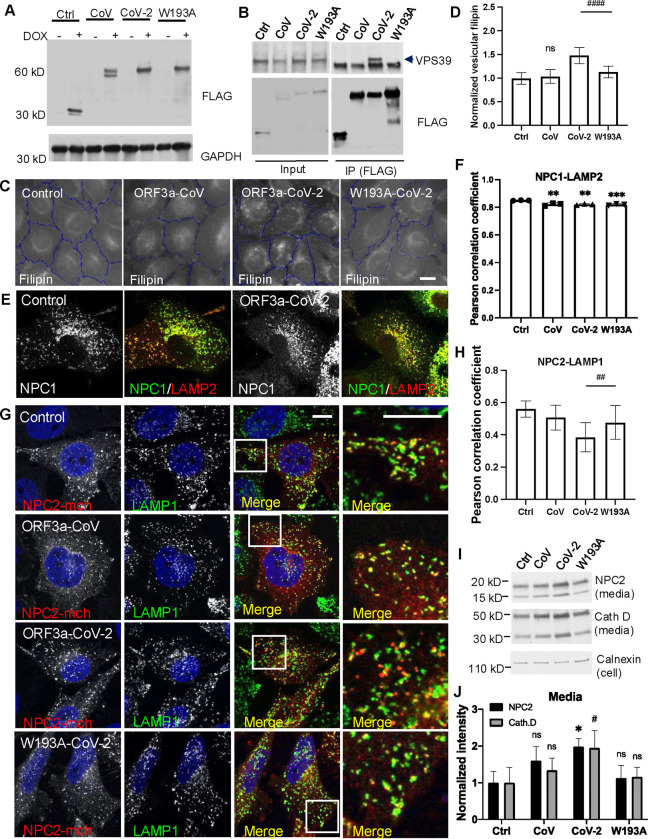
Characterization of NPC proteins in ORF3a-inducible expression cells **A.** HeLa-Flp-In cells were established as described in Methods, maintained in tetracycline-free DMEM, and treated with or without 1 *μ*g/ml doxycycline for 16 hours. Cells were lysed with 2X SDS sample buffer, and the lysates were subjected to immunoblotting to detect the protein expression with a FLAG antibody. **B.** HeLa-Flp-In cells were treated with 1 *μ*g/ml doxycycline for 16 hours and subjected to co-immunoprecipitation with FLAG antibody (M2)-coated magnetic beads. Immunoblotting was performed to detect endogenous VPS39 with an antibody. VPS39 bands were pointed by an arrow. **C,D.** HeLa-Flp-In cells were seeded in an optical 96-well plate at 8,000 cell per well, treated with 1 *μ*g/ml doxycycline for 16 hours, fixed, and stained with filipin, CellMask, and DAPI for high-content imaging. Vesicular filipin was quantified in at least 1,000 cells per well, 6 wells per experiments, 5 independent experiments. **E,F.** Antibodies against NPC1 or LAMP2 were used for immunostaining in fixed cells. Confocal microscopy and high-content imaging was performed to analyze colocalization between NPC1 and LAMP2. **G,H.** Cells were transfected with NPC2-mCherry plasmid and immunostained with antibodies of mCherry and LAMP1. Confocal microscopy was performed to image transfected cells, and FIJI was used to quantify colocalization between NPC2-mCherry and LAMP1 in the cells from two independent experiments. **I,J.** Cell culture media was collected from the HeLa Flp-In cells and subjected to immunoblotting using the antibodies indicated. Cell lysates were immunoblotted with calnexin antibody to serve as an internal control. The function of “Gels” in FIJI was applied to quantify the bands from 3 replications in each of two independent experiments. Bar graphs are presented as mean ± SD. *p* values were determined using *t’* test (D,H, CoV-2 vs W193A) or One-way ANOVA test. * or #, *p*<0.05, ** or ##, *p*<0.01, ***, p<0.001, **** (vs. control) or #### (CoV-2 vs W193A), *p*<0.0001, n.s., not significant. Scale bars, 5 *μ*m. Control (Ctrl), linker peptide, CoV, SARS-CoV ORF3a, CoV-2, SARS-CoV-2 ORF3a, W193A, SARS-CoV-2 ORF3a-W193A mutant.

With the understanding of structural basis of the interaction interface, we chose W193A mutation to block the interaction between ORF3a and VPS39. To minimize potential artifacts from overexpression, we individually introduced SARS-CoV ORF3a, SARS-CoV-2 ORF3a, the SARS-CoV-2 ORF3a W193A mutant, and a linker peptide that serves as a control into HeLa-Flp-In cells to generate tetracycline-inducible protein-expression cell lines. This system controls protein levels via a pre-engineered recombination site in the cells^44,45^. Likely due to low protein expression, we could not detect the viral proteins by immunofluorescence, but immunoblotting showed expected bands in response to induction by the tetracycline analog doxycycline (Fig. 4A). Using immunoprecipitation, we confirmed that SARS-CoV-2 ORF3a, but not SARS-CoV ORF3a, interacted with endogenous VPS39, and the W193A mutation abolished this interaction (Fig. 4B). High-content imaging quantified filipin puncta in the four cell lines, showing a ~50% increase in filipin in SARS-CoV-2 ORF3a-expressing cells compared to control cells, but not in SARS-CoV ORF3a-expressing cells (Fig. 4D). The W193A mutant significantly reduced ORF3a-induced cholesterol accumulation, although not entirely (Fig. 3D). These results aligned with findings from transiently transfected cells (Figs. 2A,B & S2B,C). Notably, the filipin levels in Flp-In cells were lower than in plasmid-transfected cells, suggesting a correlation between ORF3a expression levels and lysosomal cholesterol accumulation. Our previous study indicated that deletion of HOPS subunits in HeLa cells reduced lipid droplets due to decreased cholesteryl ester formation^19^. Consistently, reduced lipid droplets were observed only in cells expressing SARS-CoV-2 ORF3a, not in the other three lines (Figs. S4A,B). Collectively, these findings demonstrate that the Flp-In cells are suitable for mechanistic studies.

Previous research has shown that ORF3a expression increases lysosome pH^38^ and damages the lysosome membrane^46^, raising the question of whether lysosomal membrane damage or dysfunction leads to cholesterol sequestration. To investigate this, we permeabilized lysosomal membranes using the lysosomotropic agent Leu-Leu-O-Me (LLOMe) and confirmed membrane damage with the marker galectin-3^47^, observing elevated lysosomal cholesterol (Fig. S4C,D). However, deacidifying lysosomes with the ionophore monensin or V-ATPase inhibitors bafilomycin and chloroquine produced inconsistent results: monensin had limited or modest effects, while bafilomycin and chloroquine dramatically increased cholesterol (Fig. S4AC). Interestingly, SARS-CoV ORF3a, which is known to damage lysosomes^48^, did not alter cholesterol distribution (Figs. 2C,D & 4C,D). In our Flp-In cells, we observed decreased cathepsin L activity in all three ORF3a-expressing lines compared to control cells (Fig. S4E), possibly due to pH changes, but no galectin-3 puncta were detected in any line (Fig. S4F). These results suggest that lysosomal pH and membrane integrity can affect cholesterol transport, although this may depend on the extent of damage or pH change. In our Flp-In cell model, changes in pH or membrane integrity did not align with cholesterol sequestration, suggesting they were not the cause. However, we cannot rule out the possibility that lysosomal dysfunction or damage contributes to cholesterol sequestration in viral infections or other models.

We next examined the lysosomal cholesterol transporters NPC proteins in the Flp-In cells. Immunoblotting the endogenous proteins did not show any obvious difference in protein levels between the four cell lines (Fig. S4G) or between mock and infected cells (Fig. S4H). Immunofluorescence revealed that NPC1 colocalized with LAMP2 (Fig. 4E), but quantification by high-content imaging showed a slight yet statistically significant decrease in colocalization in the three ORF3a-expressing lines, as indicated by the Pearson Correlation Coefficient (Fig. 4F). Since only SARS-CoV-2 ORF3a sequestered cholesterol (Figs. 4C,D), these changes in colocalization are unlikely to explain the cholesterol transport defects observed. To investigate whether ORF3a affected NPC2 transport, we transfected the Flp-In cells with a plasmid encoding mCherry-tagged NPC2 and measured the colocalization between NPC2 and LAMP1. A mislocalization of NPC2 was observed in the SARS-CoV-2 ORF3a-expressing cells, as indicated by a significantly reduced Pearson correlation coefficient, and this mislocalization was partially rescued by the W193A mutant (Figs. 4G,H). Further analysis of NPC2 secretion revealed a significantly increased level of NPC2 in the culture media of SARS-CoV-2 ORF3a-expressing cells (Figs. 4I,J). This increased secretion was also observed for another lysosomal lumenal protein, cathepsin D (Figs. 4I,J). These results suggest that ORF3a induces a trafficking defect in NPC2, likely due to a dysregulation of general TGN-to-endosome protein transport.

### ORF3a disturbs protein endosome-to-TGN transport

**Figure 5:**
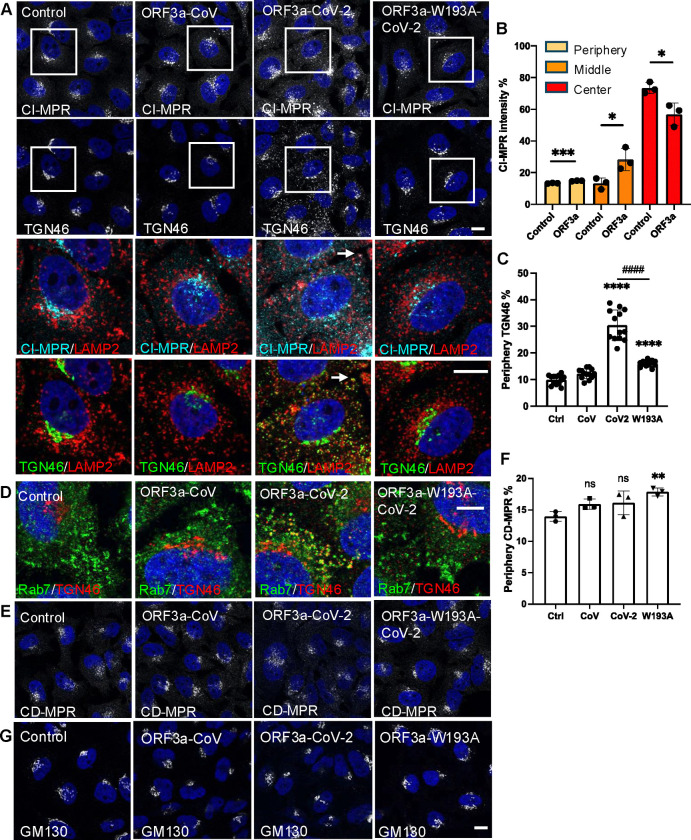
Alteration of M6PR trafficking **A-F.** HeLa Flp-In cells were fixed after 16-h induction of protein expression and immunostained with the indicated antibodies. Confocal microscopy (images) and high-content imaging (bar charts) was performed to analyze protein cellular distribution and colocalization. Periphery was defined as a ring area shrunk from cell boundary with a gap distance 15 pixels (B,C,F). Center was defined as a dilated circle from nuclear boundary with a distance of 20 pixels (B). Middle was defined as the area between periphery and center (B). **G.** Vesicular filipin was measured by high-content imaging analysis in the indicated cells. Bar graphs are presented as mean ± SD. *p* values were determined using *t’* test (B and C, CoV-2 vs W193A) or One-way ANOVA test. *, *p*<0.05, **, *p*<0.01, ***, p<0.001, **** (vs. control) or #### (C, CoV-2 vs W193A), *p*<0.0001, n.s., not significant. Scale bars, 5 *μ*m. The scale bar in D is 2 *μ*m. Control (Ctrl), linker peptide, CoV, SARS-CoV ORF3a, CoV-2, SARS-CoV-2 ORF3a, W193A, SARS-CoV-2 ORF3a-W193A mutant.

The NPC2 trafficking defect observed in SARS-CoV-2 ORF3a-expressing cells resembled that in VPS39-deleted cells, in which the sorting receptor for lysosomal lumenal proteins, CI-MPR, is abnormally degraded in lysosomes, leading to enhanced NPC2 secretion^19^. However, surprisingly, we did not observe reduced CI-MPR levels in SARS-CoV-2 ORF3a-expressing cells (Fig. S4G) or in SARS-CoV-2-infected cells (Fig. S4H). Instead, CI-MPR displayed an altered cellular distribution: it was dispersed from its usual location near the microtubule-organizing center (MTOC) to the cell periphery (Figs. 5A,B). A more severe dispersion of the TGN protein TGN46 was also observed in SARS-CoV-2 ORF3a-expressing cells (Fig. 5A,C). Normally, CI-MPR and TGN46 do not colocalize at the MTOC. However, in SARS-CoV-2 ORF3a-expressing cells, both proteins colocalized with LAMP2 at the cell periphery (Fig. 5A, arrows). Furthermore, TGN46 was found colocalized with Rab7 in vesicles at the cell periphery (Fig. 5D), suggesting that TGN46 was retained within the Rab7- or LAMP2-positive endosome-lysosome system. We also examined CD-MPR, another mannose-6-phosphate receptor involved in NPC2 transport, but observed no obvious difference in protein expression among the Flp-In cell lines (Fig. S4G). While there appeared to be a slight increase in peripheral CD-MPR, it was not statistically significant in SARS-CoV- or SARS-CoV-2 ORF3a-expressing cells (Fig. 5F), indicating that this is unlikely to contribute to the NPC2 trafficking defect. These results suggest an abnormal retrieval of proteins from endosomes to the TGN, resulting in their prolonged retention within the endosome-lysosome system. This interpretation is further supported by the unchanged localization of the Golgi-resident protein GM130 (Fig. 5G), indicating that the dispersal of CI-MPR and TGN46 was not due to Golgi unstacking.

### SARS-CoV-2 ORF3a reduced BMP

**Figure 6:**
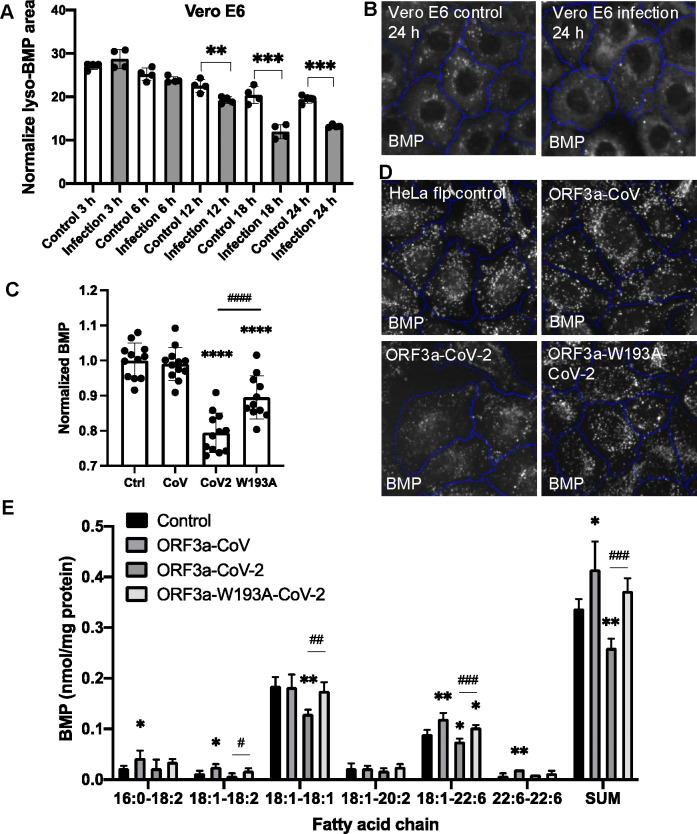
Analysis of BMP levels **A,B.** Vero E6 cells were infected with SARS-CoV-2, fixed at the indicated time points post-infection, and immunostained with BMP and LAMP1 antibodies. LAMP1-positive BMP puncta were quantified with the high-content imaging system. Representative images were shown in B. **C,D.** BMP in the HeLa Flp-In cells were measured as described in A. **E.** The indicated cells were harvested and lysed for total lipid extraction. Shotgun lipidomics was performed to identify and quantify bio(monoacylglycero)phosphate (BMP) species based on the fatty acid chains, labeled as carbon number : double-bond number. BMP was normalized to protein concentrations. **F.** BMP levels were measured as described in A in the indicated cells.

BMP, found exclusively on late endosome and lysosome membranes, physically interacts with NPC2 to facilitate cholesterol egress^29,30^. Using an antibody against BMP^26^, we observed significantly decreased BMP levels in SARS-CoV-2-infected Vero E6 cells, starting 12 hours post-infection (Figs. 6A,B). The accumulation of lysosomal cholesterol in infected cells was detected 18 hours post-infection, following the initial BMP reduction (Fig. 1D), suggesting that impaired cholesterol egress may be attributed to decreased BMP. To determine whether ORF3a changes BMP, we further examined the HeLa-Flp-In cells and found that BMP levels were reduced by 20% in the SARS-CoV-2-ORF3a cells, compared to the control cells, while the W193A mutation partially rescued this reduction (Figs. 6C,D). The SARS-CoV-ORF3a cells did not show a changed level of BMP (Figs.6C,D). To validate these findings, we extracted total lipids from the four Flp-In cell lines and performed shotgun lipidomics analysis. BMP levels, normalized to protein content, were quantified across six detectable species. The two most abundant species, 18:1–18:1 and 18:1–22:6 BMP, were reduced by approximately 20% in SARS-CoV-2 ORF3a-expressing cells compared to controls, with levels restored in W193A cells (Fig. 6E). We used complementary methods and cell models to demonstrate BMP reduction due to either SARS-CoV-2 infection or ORF3a expression. This BMP decrease aligned with increased cholesterol levels in SARS-CoV-2 ORF3a-expressing cells, partially rescued in W193A cells, while no change was observed in SARS-CoV ORF3a-expressing cells (Figs. 4C,D). These findings suggest a correlation between alterations in BMP levels and cholesterol accumulation in lysosomes, and that BMP reduction may represent an additional mechanism underlying SARS-CoV-2-induced cholesterol sequestration.

### Implications of Cholesterol Sequestration for SARS-CoV-2

**Figure 7:**
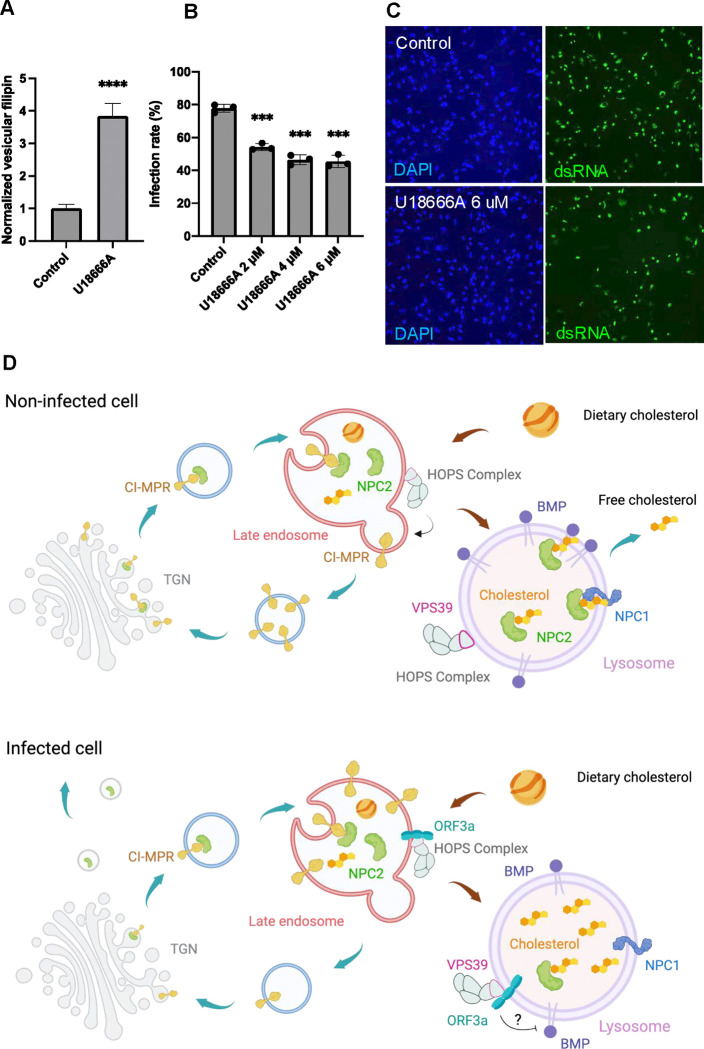
Implications of cholesterol sequestration in SARS-CoV-2 **A.** A549-hACE2 cells were treated with or without 6 *μ*M U18666A for 2 h, and filipin intensity was measured using the high-content imaging system. **B,C.** A549-hACE2 cells were pre-treated with U18666A at the indicated concentrations for 2 h and subjected to SARS-CoV-2 infection. After 24 h, the cells were fixed and immunostained with an antibody against dsRNA to identify infected cells. Nuclei were stained with DAPI to identify total cell numbers. High-content imaging system was used to image and quantify the infection rates, calculated as infected cell number/total cell number %. **D.** Scheme of the pathways participating in lysosomal cholesterol egress. In healthy cells, lysosomes process endocytosed low-density lipoproteins (LDLs) to release free cholesterol, which is exported via the cholesterol transporters Niemann-Pick C 2 (NPC2) and NPC1. NPC2 also binds with the phospholipid bis(monoacylglycero)phosphate (BMP) to export cholesterol independently of NPC1. Newly synthesized NPC2 associates with the sorting receptor mannose-6-phosphate receptors, such as CI-MPR, in the trans-Golgi network (TGN) and is delivered to late endosomes. Here, CI-MPR is retrieved and recycled back to the TGN with help from the HOPS complex. In SARS-CoV-2-infected cells, ORF3a interacts with the HOPS subunit VPS39, disrupting CI-MPR recycling. This leads to NPC2 trafficking defects, i.e., mislocalization and increased secretion. Additionally, the ORF3a-VPS39 interaction reduces BMP levels by an undefined mechanism, together resulting in cholesterol sequestration within lysosomes.

Cholesterol sequestration in lysosomes likely disrupts cellular cholesterol homeostasis, leading to broader defects in lipid metabolism. But what does this mean for the virus? Based on our findings and a previous study^11^, we hypothesize that cholesterol accumulation in lysosomes reduces cholesterol levels at the plasma membrane, potentially impacting SARS-CoV-2 cell entry. This may serve as a viral strategy to limit secondary infection within the same cell, thereby preventing viral overload. To test this hypothesis, we used the NPC1 inhibitor U18666A^49^ in A549-hACE2 cells to mimic SARS-CoV-2-induced cholesterol egress defects, then infected these treated cells with the virus. As expected, U18666A increased lysosomal cholesterol (Fig. 7A). Notably, infection quantification showed that U18666A treatment significantly decreased SARS-CoV-2 infection compared to control cells (Figs. 7B,C). These results support our hypothesis that SARS-CoV-2 manipulates host cholesterol distribution to limit secondary infections for its benefit. This hypothesis is further supported by the observed downregulation of the entry receptor ACE2 after infection (Fig. S4H) and decreased BMP levels (Figs. 6A–E), both critical components in SARS-CoV-2 infection. These changes may represent coordinated viral mechanisms to optimize host cell environments for their replication.

## DISCUSSION

Our study demonstrates that SARS-CoV-2 infection sequesters cholesterol within lysosomes and reduces the levels of bis(monoacylglycerol)phosphate (BMP) levels, along with the corresponding molecular mechanisms. Given BMP promotes lysosomal cholesterol egress, the reduction of BMP could enhance cholesterol sequestration and further interrupts cholesterol homeostasis, including cholesterol biosynthesis, uptake, transport, and clearance. This manipulation of lysosomal lipid composition presents a potential mechanism underlying the dysregulated cholesterol metabolism observed in COVID-19 patients, including those with long COVID. This work provides cell-level mechanistic insight into how SARS-CoV-2 disrupts cholesterol homeostasis, offering a potential explanation for the clinical abnormalities reported in COVID-19 cases.

Lysosomal cholesterol accumulation and BMP depletion may have broader implications in COVID-19 pathology beyond dysregulated lipid metabolism. Both cholesterol and BMP are key players in lysosomal membrane integrity and function, which are critical for cellular homeostasis. An imbalance in these lipids could contribute to the organelle dysfunction observed in SARS-CoV-2 infection, potentially affecting immune responses, autophagy, and cellular energy regulation, as well as contributing to the persistent symptoms seen in long COVID. Thus, targeting lysosomal cholesterol handling may represent a novel therapeutic avenue for managing COVID-19-related metabolic disturbances.

The interaction of SARS-CoV-2 protein ORF3a with VPS39 appears central to these disruptions. By binding to VPS39, ORF3a alters the function of the HOPS complex, which is known to play an essential role in autophagy^50^, endosomal trafficking^51^, and lysosomal cholesterol egress^19^. This ORF3a-VPS39 interaction appears to drive the observed defects, as evidenced by our findings that introducing a point mutation to block the interaction mostly rescued cholesterol sequestration and BMP reduction in lysosomes. This insight not only expands our understanding of ORF3a’s role in cellular lipid handling but also highlights VPS39 and the broader HOPS complex as regulators of lipid metabolism.

Notably, SARS-CoV-2 can infect hepatocytes^52^, a primary cell type in the liver, which is the central organ for lipid metabolism. Although we did not directly examine hepatic cells in this study, the conserved function of the HOPS complex in lysosomal trafficking suggests that similar lipid imbalances may occur in liver cells. Such disruptions could have far-reaching effects on systemic lipid metabolism, especially given the liver’s role in managing cholesterol, lipoprotein processing, and bile acid synthesis. Future work will extend this line of investigation to liver cells, with an emphasis on lipoprotein biology, to explore the potential systemic impacts of SARS-CoV-2 on lipoprotein abnormalities observed in COVID-19 and long COVID.

Interestingly, while SARS-CoV-2 relies on cell surface cholesterol and endosomal BMP to complete its life cycle, our findings indicate that infected cells experience disrupted cholesterol distribution and reduced BMP levels. Additionally, SARS-CoV-2 infection has been shown to trigger degradation of ACE2, its own receptor, soon after entry into host cells. These alterations appear counterintuitive, as they would theoretically limit further viral entry and replication in the same cell. However, we propose that this may be a self-regulating mechanism to prevent excessive viral load within individual host cells, potentially avoiding premature cell death or triggering strong antiviral responses that could hinder the virus’s overall replication strategy. Similar mechanisms have been observed in other viruses; for example, HIV downregulates CD4 ^53–55^ in infected cells to avoid superinfection. Influenza A virus also modulates host cell metabolism in ways that initially support viral replication but then limit further infection in highly infected cells^56^. This controlled modulation by SARS-CoV-2 might be a strategic adaptation to sustain viral spread at a broader level within the host, balancing infection and cell viability. Further research is needed to determine if SARS-CoV-2 actively induces such protective changes or if they are secondary consequences of viral protein interactions, but this could represent a crucial aspect of the virus’s evolutionary strategy to optimize infection dynamics.

In summary, our findings suggest mechanisms by which SARS-CoV-2 infection disrupts host cell lipid metabolism through lysosomal cholesterol sequestration. By uncovering the functional interaction between ORF3a and VPS39, we have identified a viral-host interaction that could serve as a potential target to address the lipid dysregulation associated with COVID-19.

## MATERIALS AND METHODS

### Cells and virus

A549 and HeLa cells were cultured in Dulbecco’s Modified Eagle’s Medium (DMEM) supplemented with 10% heat-inactivated fetal bovine serum (FBS), 25 mM HEPES, and MycoZap Plus-CL (Lonza, Basel, Switzerland) at 37°C with 5% CO_2_. Vero E6 cells (ATCC, CRL-1586) were cultured in DMEM supplemented with 10% heat-inactivated FBS, 1% pen/strep, 2mM L-glut, 1% non-essential amino acids, 1% HEPES. Severe acute respiratory syndrome coronavirus 2 (SARS-CoV-2, Isolate USA-WA1/2020) was acquired from BEI Resources (NR-52281) and propagated in Vero E6 cells for 3 days. Infectious virus was isolated by harvesting cellular supernatant and spinning at 1000 g for 10 minutes to remove cellular debris and stored at −80°C. SARS-CoV-2 infectious particles were quantified in media by standard plaque assay^57^

### SARS-CoV-2 infections

Coverslips or optic 96-well plates (Agilent, Santa Clara, CA) were coated with collagen solution (0.03–0.04 mg/ml collagen in 0.02 N acetic acid) for > 30 min and then washed twice with PBS before receiving cells. A549-hACE2 cells were seeded on coverslips at 0.1 X 10^6^ cells per well or in optical 96-well plates at 0.012 X 10^6^ cells per well and infected with SARS-CoV-2 in DMEM (4% FBS) at MOI: 4 and incubated at 37°C for 1 hr on the next day. Cells were then washed twice with 1x PBS and normal culture media was added. At the indicated times post-infection, cells were fixed with 4% PFA. Vero E6 cells were seeded in optical 96-well plate around 0.025 X 10^6^ cells per well and infected with SARS-CoV-2 at MOI: 0.1 in 2% FBS media. The control group of cells were incubated with the media only.

### Generation of flip-in cells

HeLa Flp-In host cells (Thermo Fisher Scientific, Waltham, MA) were transfected with the plasmids encoding FLAG-tagged proteins or linker peptide and subjected to hygromycin selection after 48 h post transfection in DMEM as described above but replacing regular FBS with tetracycline-free FBS (Thermo Fisher Scientific) (tet-free media). Following 2-week selection, transformants were kept in tet-free medium for another 12 days to allow single colony formation. Protein expression was induced by addition of 1 *μ*g/ml doxycycline for 16 hours. The colonies had relatively low expression levels of FLAG were chosen and pooled for experiments, determined by immunoblotting of FLAG.

### Immunofluorescence, Filipin staining, and confocal microscopy

Fixed cells were washed 3 times with PBS, and primary antibodies were diluted in 1% BSA in PBS supplemented with 0.2% saponin and applied to cells for 1 h at 37°C or overnight at 4°C. Alexa-conjugated secondary antibodies (Thermo Fisher Scientific) were applied with the same saponin-contained BSA-PBS solution for 30 min at 37°C. Coverslips were mounted with DAPI-contained mounting reagents (Electron Microscopy Sciences, Hatfield, PA) and allowed to dry at 37°C for at least 1 h. To stain free cholesterol, 25 *μ*g/ml filipin complex (Millipore Sigma, St. Louis, MO) in PBS was applied to fixed cells at 37°C for 30 min. When immunostaining was performed with filipin, primary antibodies and Alexa-conjugated secondary antibodies along with DRAQ7 (Novus Biologicals, Centennial, CO) were sequentially applied to filipin-stained cells without detergent permeabilization. Then cells were mounted with mounting reagents without DAPI (Electron Microscopy Sciences) at 37°C for at least 1 h. Images were acquired using a Zeiss LSM800 confocal microscope equipped with the software ZEN (Zeiss, Oberkochen, Germany).

### High-content imaging and image analysis

Cells in optical 96-well plates were fixed and stained as described above and maintained in PBS for immediate imaging. Imaging and analysis were performed using the CellInsight Microscope platform (Thermo Fisher Scientific). DAPI- or DRAQ7-stained nuclei were scanned to determine focus through the programmed autofocus function. Cytoplasmic signals from HCS CellMask Near-IR Stain (Thermo Fisher Scientific) were applied to define individual cell boundaries. Cells were excluded based on criteria such as incomplete cell boundaries at image edges, abnormal size or shape, and signs of cell death.

For cellular punctum analysis, the cell boundary-enclosed areas are defined as ROI_A. Filipin, LAMP, or BMP puncta were detected as spots (2 pixes in size) using the software “spot detection” function. Line-shaped filipin signals from the plasma membrane were excluded by setting the validation criteria for shape. Puncta that overlapped with ROI_A were quantified by their fluorescence intensity. To measure colocalized signals, the software function of “colocalization” was applied, and the output feature “ROI_A(B) Correlation Coefficient” was extracted for the quantification of Pearson Correlation Coefficient.

To analyze the infected cells, dsRNA was immunostained with an antibody, and its signals were used to set the threshold for detection of infected cells, using the function “Reference” of the software.

### Cholesterol measurement by GC-MS

Cells were collected in PBS with a cell scraper after washing twice with PBS, and the total lipids were extracted by adding lipid-extraction solvent (chloroform:methanol:acetic acid = 50:50:1) of 90% volume of PBS. The organic phase and aqueous phase were separated by 10 minute-centrifugation at 21,000 g. Organic phase was collected and transferred into a new tube and dried with vacuum. The extracted lipids were stored at −80°C for further analysis. Cholesterol calibration standard solutions were prepared from the concentration of 50 *μ*g/ml to 400 *μ*g/ml along with 80 *μ*g/ml 5a-cholestane as the internal standard. The linear calibration curve was obtained with R2 =0.9884. The dried lipids were dissolved with hexane and mixed with 4 *μ*g 5a-cholestane. The GC-MS analysis was conducted on Agilent 6890/5973 with selected ion monitoring (SIM) mode.

### Shotgun Lipidomics

Lipid species were analyzed using a multidimensional mass spectrometry-based shotgun lipidomics approach^58^. In brief, each cell sample homogenate containing 0.3 mg of protein which was determined with a Pierce BCA assay was accurately transferred to a disposable glass culture test tube. A premixture of lipid internal standards was added prior to conducting lipid extraction for quantification of the targeted lipid classes. Lipid extraction was performed using a modified Bligh and Dyer procedure (Wang and Han 2014), and each lipid extract was reconstituted in chloroform:methanol (1:1, v:v) at a volume of 400 *μ*L/mg protein. Derivatization of lipid extracts for analysis of free fatty acid (FFA) and bis(monoacylglycero)phosphate (BMP) was performed as described previously^59,60^ before lipidomics analysis. Lyso-phosphatidylglycerol (LPG) in the aqueous phase was enriched using a HybridSPE cartridge. After washing with methanol, lysophospholipids were eluted with methanol/ammonia hydroxide (9:1), dried and reconstituted in methanol for lipidomics analysis ^61^. For shotgun lipidomics, individual lipid samples prepared as aforementioned was further diluted to a final concentration of ~500 fmol total lipids per *μ*L. Mass spectrometric analysis was performed on a triple quadrupole mass spectrometer (TSQ Altis, Thermo Fisher Scientific, San Jose, CA) and a Q Exactive mass spectrometer (Thermo Scientific, San Jose, CA), both of which were equipped with an automated nanospray device (TriVersa NanoMate, Advion Bioscience Ltd., Ithaca, NY) as described^62^. Identification and quantification of lipid species were performed using an automated software program^63,64^. Data processing (e.g., ion peak selection, baseline correction, data transfer, peak intensity comparison, and quantitation) was performed as described^64^. The results were normalized to the protein content (nmol lipid/mg protein).

### Analysis of intracellular and extracellular proteins by Western blotting

To detect secreted NPC2 and cathepsin D, cells were kept in a minimum volume of DMEM without serum for 6 h after three-time washing with the same medium. The media were collected and centrifuged at 1,000 g for 5 min to pellet any detached cells. The supernatants were mixed with SDS loading buffer and subjected to SDS-PAGE and immunoblotting. After collecting the media, cells were incubated in complete DMEM for 2 h. Cell lysates were obtained by using 2X SDS sample buffer.

### Quantification and statistical analysis

Image J was used for quantifying fluorescence signals of randomly-taken images from at least three independent experiments. The signal intensity of vesicular filipin was measured using the function of “Analyze particles”, and co-localization was analyzed with the plug-in “PSC Colocalization”^83^. High-content imaging results were obtained from more than 1,000 cells per well and at least 3 wells per experiment. The number of trials is specified in the legends where appropriate. The bar graphs show the pooled results displayed as mean ± SD. Colocalization results include frequency distribution. Statistical significance was determined by comparing two datasets using a *t* test with one-tailed distribution or more than two datasets using one-way ANOVA, using the software Prism 9 (GraphPad). Probability values and number of trials are given in the figure captions and the legends where appropriate. The statistical significance is generally denoted as follows: *, *p* < 0.05, **, *p* < 0.01, ***, *p* < 0.001, ****, *p* < 0.0001, and n.s., not significant.

## Supplementary Material

Supplement 1
