# Supplementary material for: SARS-CoV-2 ORF3a blocks lysosomal cholesterol egress by disrupting VPS39-regulated NPC2 trafficking and BMP metabolism
Source: bioRxiv. 2025 Sep 20:2024.11.13.623299. Originally published 2024 Nov 14. Preprint. [Version 2] doi: 10.1101/2024.11.13.623299 (PMC11601339; doi:10.1101/2024.11.13.623299)
Supplement: Supplement 1 [file media-1.pdf]

SUPPLEMENTAL INFORMATION

Supplemental figures

Figure S1

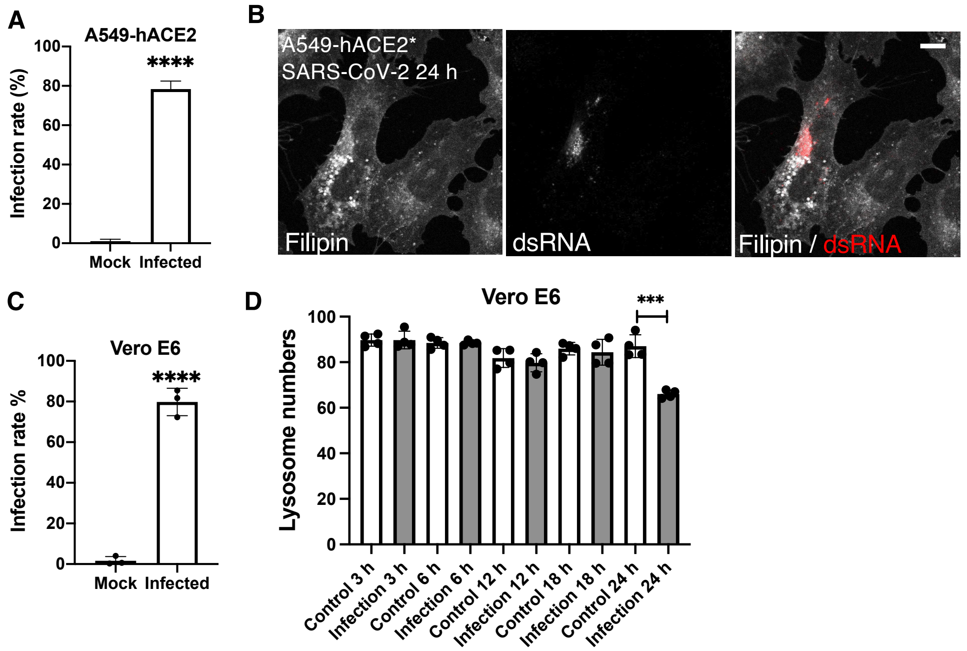

Fig. S1. SARS-CoV-2 infection sequestrates cholesterol in lysosomes

A. A549-hACE2 cells were infected with SARS-CoV-2, and the infection rate was determined by immunostaining of dsRNA and quantified using a high-content imaging system. **B.** An additional line of A549 cells stably expressing human ACE2 (A549-hACE2*) were infected with SARS-CoV-2, fixed at 24 h post-infection, stained with the antibodies against dsRNA and filipin, and imaged with a confocal microscope. Note that the dsRNA-positive cell showed increased filipin intensity, compared to the dsRNA-negative cell. Scale bars, 5 μm. **C.** Vero E6 cells were infected with SARS-CoV-2, and the infection rate was determined by immunostaining of dsRNA and quantified by high-content imaging system. D. Vero E6 cells were infected with SARS-CoV-2, fixed, immunostained with a LAMP1 antibody, and analyzed by high-content imaging. Bar graphs are presented as mean ± SD. *p* values were determined using *t* test. ***, $p<0.001$. ****, $p<0.0001$.

Figure S2

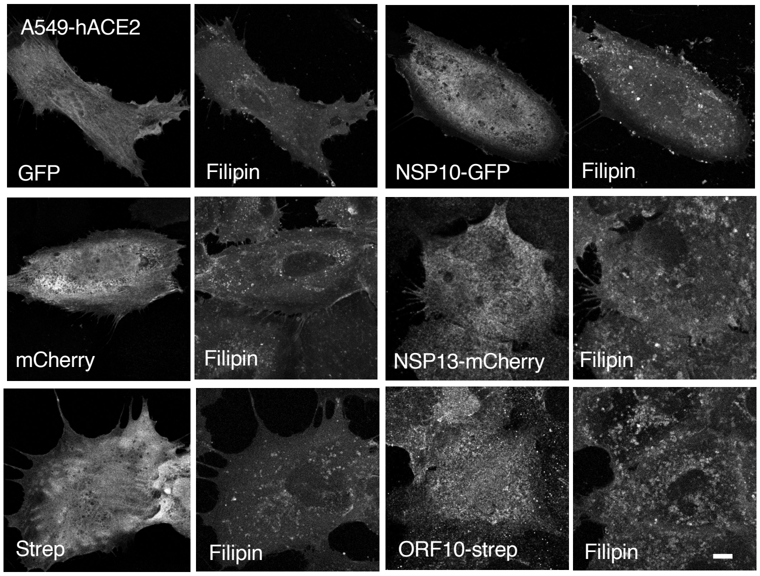

Fig. S2. Cellular localization of NSP10, NSP13, and ORF10

A549-hACE2 cells were transfected with the plasmids that encode NSP10, NSP13, or ORF10 with a different tag. Cells were fixed at 24 h post-transfection, stained with filipin and the antibodies against GFP, mCherry, or strep, and imaged with a confocal microscope. Scale bars, 10 μm.

Figure S3

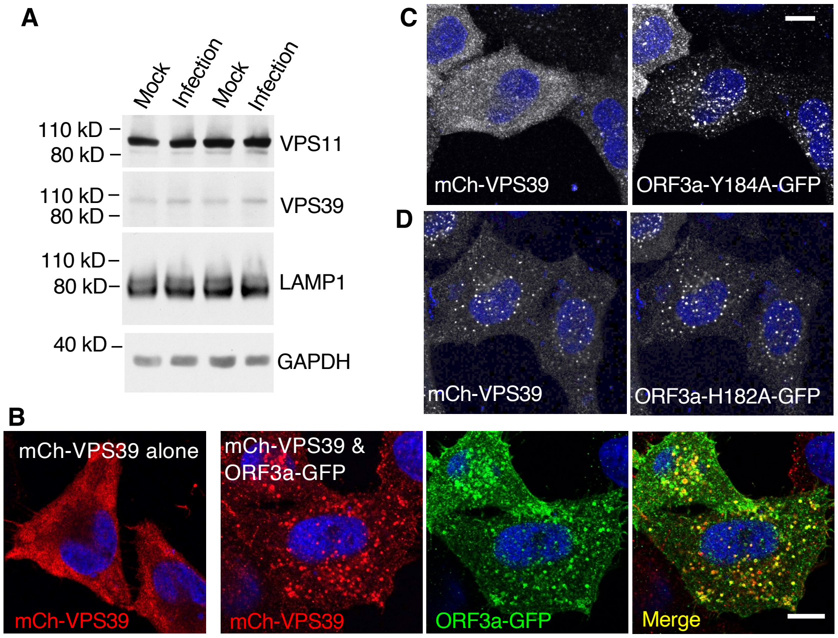

Fig. S3. Characterization of ORF3a-VPS39 interaction

A. A549-hACE2 cells were infected with SARS-CoV-2 and lysed at 24 h post-infection. Cell lysates were subjected to immunoblotting with the indicated antibodies. **B-D.** HeLa cells were transfected with mCh-VPS39 construct alone (**B, left**) or co-transfected with ORF3a-GFP (**B, right**) or ORF3a mutant constructs (**C,D**). The cells were fixed at 24 h post transfection, immunostained with the antibodies against GFP and mCherry, and imaged with a confocal microscope. Note that mCh-VPS39 alone displayed cytosolic distribution (**B, left**). When ORF3a was present, mCh-VPS39 formed puncta, colocalized with ORF3a (**B, right**). This distribution alteration was used as an indicator of ORF3a-VPS39 interaction in examining those ORF3a mutants (**C,D**). Scale bars, 5 µm.

Figure S4

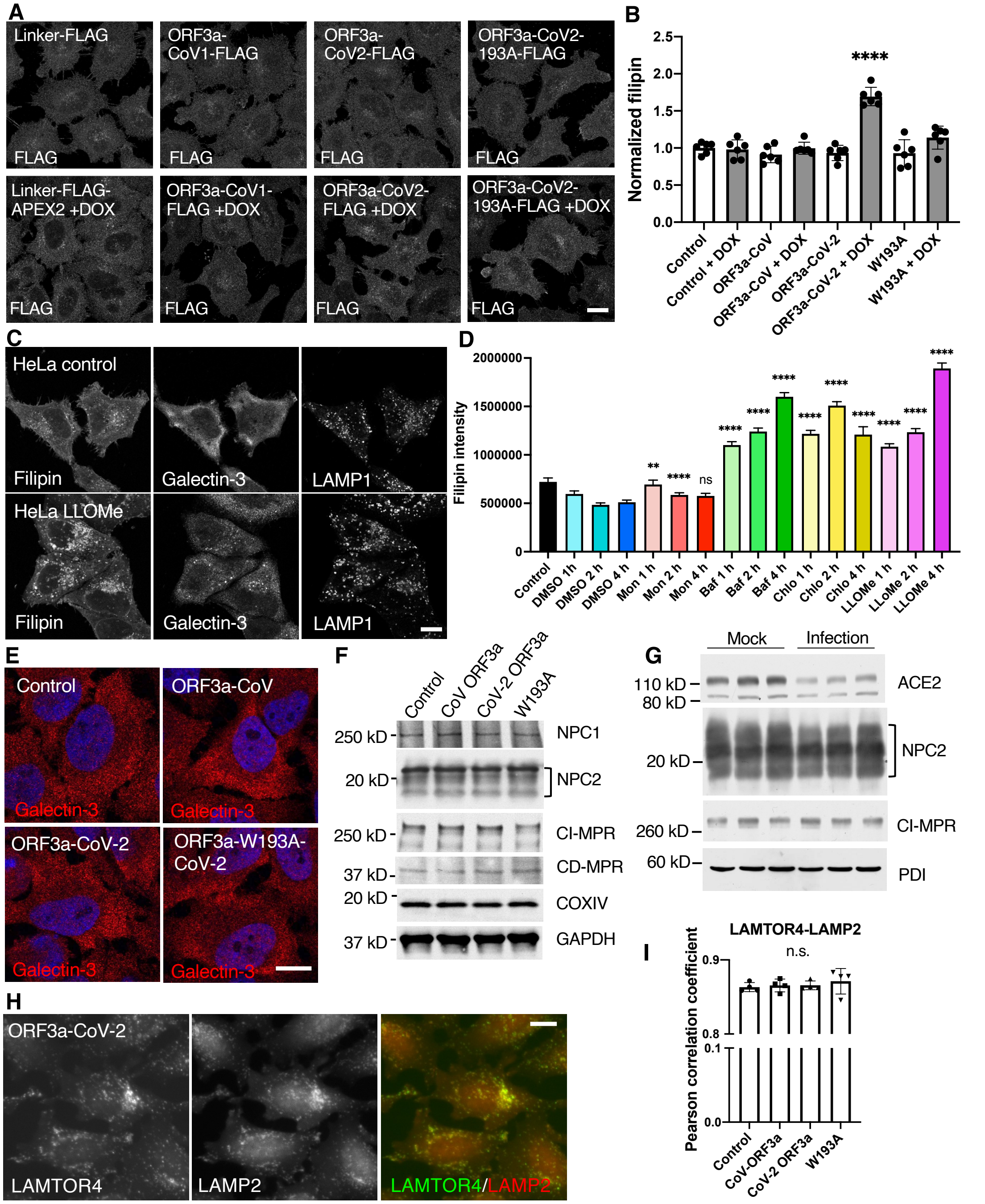

Fig. S4. The impacts of ORF3a-VPS39 interaction on lysosome integrity

A. HeLa Flp-In cells were treated with or without doxycycline (DOX), fixed, stained with a FLAG antibody, and imaged with a confocal microscope. **B**. The Flp-In cells were treated with or without doxycycline, fixed, stained with filipin, and analyzed by high-content imaging for filipin signals. **C.** HeLa cells were treated with 0.5 mM LLOMe for 1

h, fixed, stained with filipin and a galectin-3 antibody, and imaged with a confocal microscope. **D.** HeLa cells were treated with 10 μM monensin, 100 nM bafilomycin, 100 μM chloroquine, or 0.5 mM LLOMe for the indicated time periods, fixed, stained with filipin, and analyzed with high-content imaging. 0.1% DMSO in cell culture media served as a vehicle control. **E.** Example confocal microscopy images of HeLa Flp-In cells, immunostained with a galectin-3 antibody. Cell lysates from HeLa Flp-In cells (**F**) or SARS-CoV-2-infected A549-hACE2 cells (24 h post-infection, **G**) were subjected to immunoblotting with the indicated antibodies. **H,I.** HeLa Flp-In cells were immunostained with LAMTOR4 and LAMP2 antibodies and analyzed by high-content imaging. The colocalization between LAMTOR4 and LAMP2 was quantified and presented as Pearson correlation coefficient. Bar graphs are presented as mean ± SD. p values were determined using one-way ANOVA. **, $p < 0.01$. ****, $p < 0.0001$. n.s., no significant difference. Scale bars, 5 μm.

Figure S5

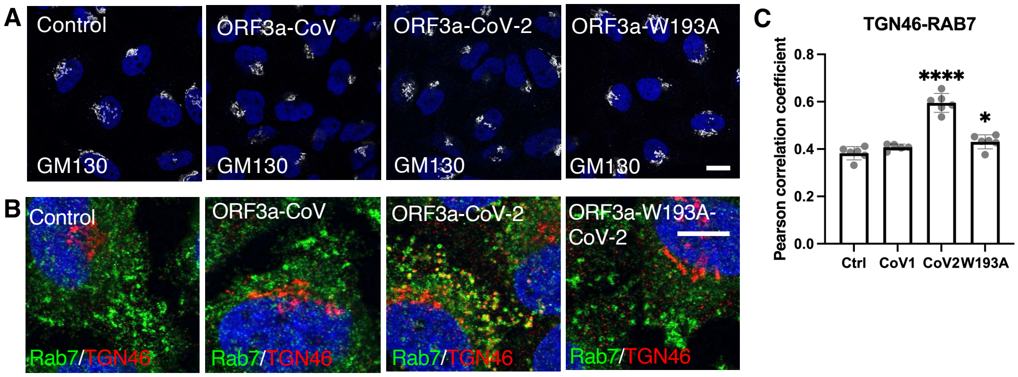

Fig. S5. Characterization of Trans-Golgi Network (TGN) proteins

HeLa Flp-In cells were fixed, immunostained with the indicated antibodies. Images were taken either with a confocal microscope **(A,B)** or high-content imaging system for quantification of the colocalization between TGN46 and Rab7 **(C).** Scale bars, 5 µm. Bar graphs are presented as mean ± SD. *p* values were determined using one-way ANOVA test. ****, $p < 0.0001$.

Figure S6

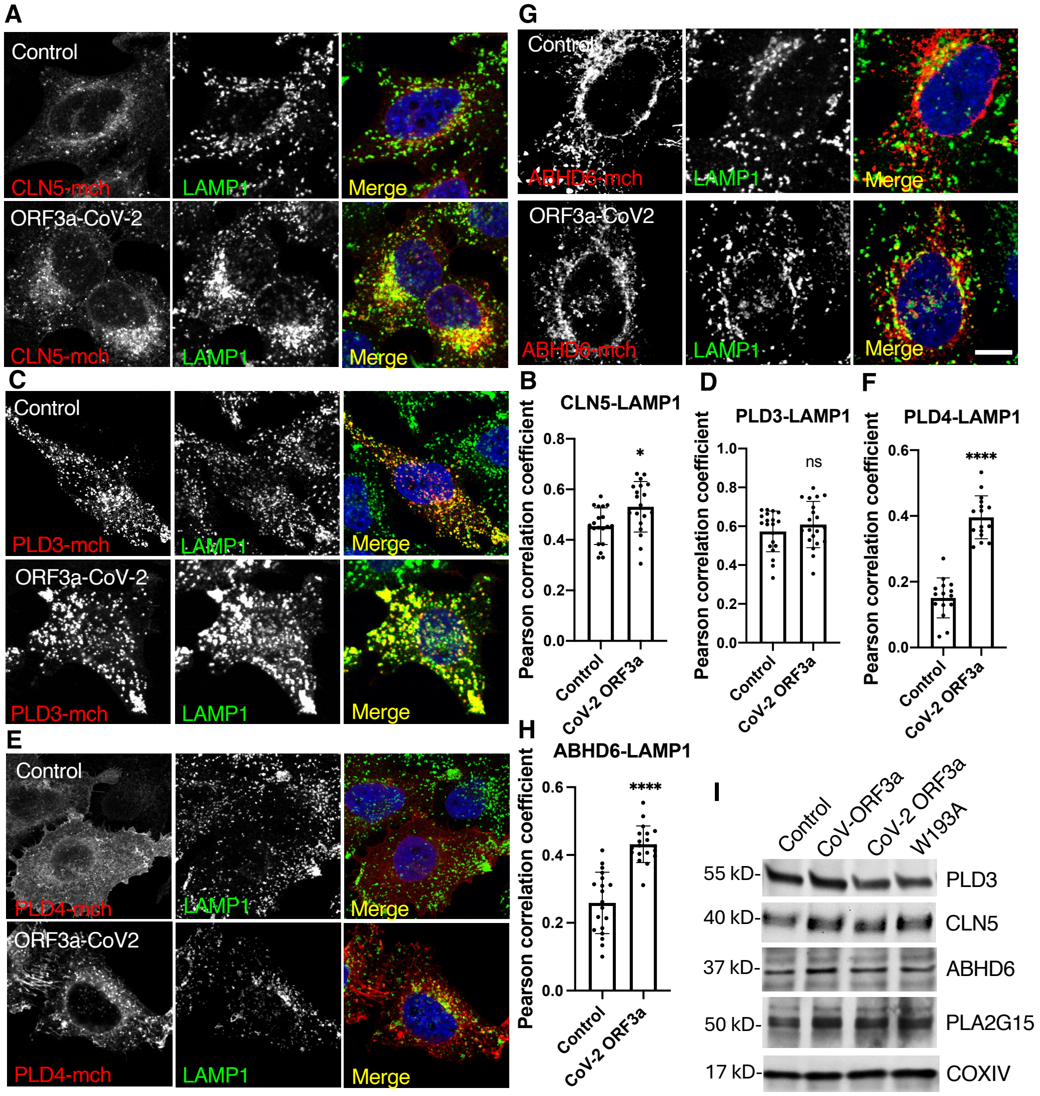

Fig. S6. Characterization of BMP enzymes

HeLa Flp-In control and CoV-2-ORF3a cells were transfected with the plasmids encoding BMP synthesis or turnover enzymes, fixed at 24-h post-transfection, immunostained with mCherry and LAMP1 antibodies. Images were taken with a confocal microscope **(A,C,E,G)** and used for quantification of the colocalization between the enzymes and LAMP1 by FIJI **(B,D,F,H).** Scale bar, 5 µm. **I.** Cell lysates from HeLa Flp-In cells were subjected to immunoblotting with the indicated antibodies. Bar graphs are presented as mean ± SD. *p* values were determined using *t* test. *, $p < 0.05$. ****, $p < 0.0001$. n.s., no significant difference.

Figure S7

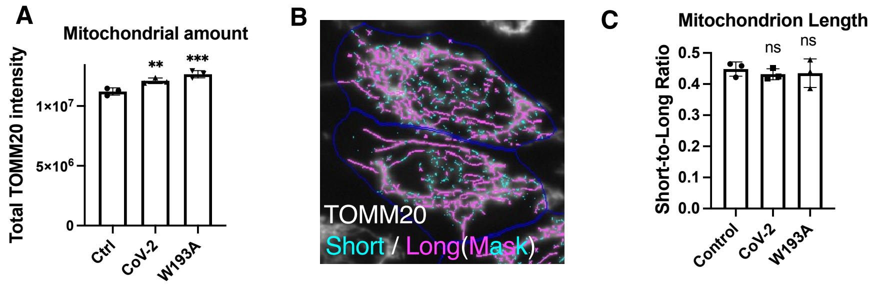

Fig. S7. Mitochondrial quantifications

HeLa Flp-In cells were fixed, immunostained with a TOMM20 antibody, and analyzed with high-content imaging. Average total intensity of TOMM20 (**A**) and the short-to-long ratio of mitochondria (**C**) were quantified. Short mitochondria were defined as those with a length shorter than 1 μm (**B**).

Figure S8

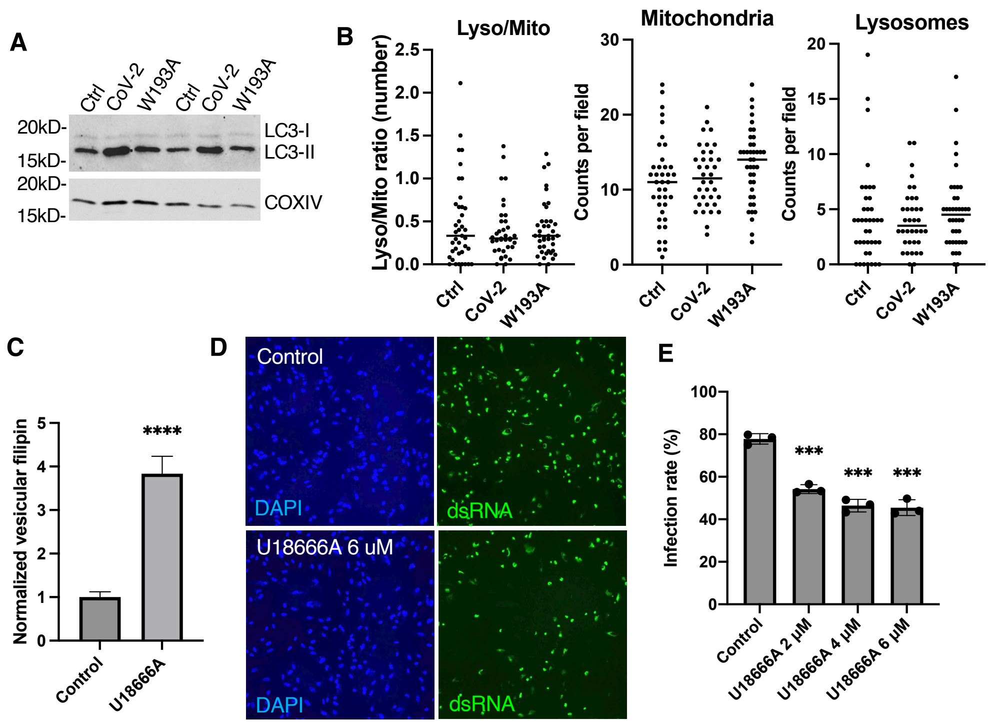

Fig. S8. Quantification of lysosome-mitochondrion membrane contact sites and the effect of lysosomal cholesterol on SARS-CoV-2 infectivity

A. Cell lysates from HeLa Flp-In cells were subjected to immunoblotting with the indicated antibodies. **B.** HeLa Flp-In cells were fixed and imaged by an electron microscope. The numbers of lysosomes and mitochondria were quantified from 40 images of 20 cells of each cell type. **C**. A549-hACE2 cells were treated with 2 μM U18666A for 4 h, fixed, stained with filipin, and quantified for filipin signals with high-content imaging. **D,E.** A549-hACE2 cells were treated with U18666A at the indicated concentration for 4 h and infected with SARS-CoV-2. Control cells were treated with the same amount of DMSO as in 6 μM U18666A for 4 h and infected equally. Cells were fixed at 24 h post-infection and immunostained with a dsRNA antibody to identify the infected cells. DAPI-stained nuclei were used to identify total cell numbers.

Supplemental tables

Table. S1 cDNA constructs used in this study

**Insert**	**Vector**	**Tag**	**Remarks**
ABHD6	pmCherry-N1	c-mCherry	This study
CLN5	pmCherry-N1	c-mCherry	This study
GFP-E	pEGFPC1	n-GPF	Addgne # 165123
GFP-M	pEGFPC1	n-GFP	Addgene #165124
Linker peptide	pDEST-Flp-In	c-FLAG-APEX2	This study
NPC2	mcherry-N1	c-mCherry	Gift from Dr. P. Lobel (Rutgers University)
SARS-CoV2-NSP1-strep	pLVX-EF1alpha-IRES-Puro	c-Strep	Addgene #141367
NSP2-strep	pLVX-EF1alpha-IRES-Puro	c-Strep	Addgene #141368
NSP3-GFP	pEGFP-N1	c- GFP	Addgene #165108
NSP4-mch	pmCherry-N1	c-mcherry	Addgene #165132
NSP5-strep	pLVX-EF1alpha-IRES-Puro	c-Strep	Addgene #141370
NSP6-mch	pmCherry-N1	c-mcherry	Addgene #165133
GFP- NSP7	pEGFP-C1	n-GFP	Addgene #165112
NSP8-GFP	pEGFP-N1	c-GFP	Addgene #165113
NSP9-strep	pLVX-EF1alpha-IRES-Puro	c-strep	Addgene #141375
NSP10-GFP	pEGFP-N1	c-GFP	Addgene #165115
NSP11-strep	pLVX-EF1alpha-IRES-Puro	c-Strep	Addgene #141377
GFP-NSP12	pEGFP-C1	n-GFP	Addgene #165117
NSP13-mch	pmCherry-N1	c-mcherry	Addgene #165136
NSP14-mch	pmCherry-N1	c-mcherry	Addgene #165137
NSP15-GFP	pEGFP-C1	n-GFP	Addgene #165120
ORF3a-CoV-2-GFP	pEGFP-N1	c-EGFP	This study
ORF3a-CoV-2-V5			This study
CoV-ORF3a	pDEST-Flp-In	c-FLAG-APEX2	This study
CoV-2-ORF3a-S171A	pEGFP-N1	c-EGFP	This study
CoV-2-ORF3a-Y184A	pEGFP-N1	c-EGFP	This study
CoV-2-ORF3a-H182A	pEGFP-N1	c-EGFP	This study
CoV-2-ORF3a	pDEST-Flp-In	c-FLAG-APEX2	Previous study
CoV-2-ORF3a-W193A	pDEST-Flp-In	c-FLAG-APEX2	This study
CoV-2-ORF3a-W193K	pEGFP-N1	c-EGFP	This study
CoV-2-ORF3a-W193H	pEGFP-N1	c-EGFP	This study

CoV-2-ORF3a-W193D	pEGFP-N1	c-EGFP	This study
CoV-2-ORF3a-W193F	pEGFP-N1	c-EGFP	This study
ORF3a-CoV-GFP	pEGFP-N1	c-GFP	Addgene # 165121
ORF3b-strep	pLVX-EF1alpha-IRES-Puro	n-strep	Addgene #141384
ORF6-strep	pLVX-EF1alpha-IRES-Puro	c-strep	Addgene #141387
ORF7a-strep	pLVX-EF1alpha-IRES-Puro	c-strep	Addgene #141388
ORF7b-strep	pLVX-EF1alpha-IRES-Puro	c-strep	Addgene #141389
ORF8-GFP	pmCellFree_KA1	n-GFP	Addgene 169395
GFP-ORF9b	pEGFP-C1	n-GFP	Addgene #165122
ORF10-strep	pLVX-EF1alpha-IRES-Puro	c-Strep	Addgene #141394
PLD3	pmCherry-N1	c-mCherry	This study
PLD4	pmCherry-N1	c-mCherry	This study
Spike	pGBW-m4137386	No tag	Addgene 149540
N-strep	pLVX-EF1alpha-IRES-Puro	c-Strep	Addgene #141391
VPS39	pmCherry-C1	n-mcherry	Previous study

Table. S2 Antibodies used in this study

Antibody	Source	Product Number	Dilution	Applications
ABHD6	Protein Tech	20494-1-AP	1:1000	WB
ACE2	Cell signaling technologies	4355	1:1000	WB
ATG5	Protein Tech	10181-2-AP	1:1000	WB
ATG7	ThermoFisher Scientific	MA5-32221	1:1000	WB
Calnexin	Cell Signaling Technologies	2679s	1:8000	WB
Cathepsin D	Protein Tech	219361	1:4000	WB
CD-MPR	Developmental Studies Hybridoma Bank	22d4	1:50	WB
CI-MPR	Abcam	124767	1:500	IF
CI-MPR	Protein Tech	20253-1-AP	1:1000/1:500	WB/IF
CLN5	Abcam	ab170899	1:1000	WB
COXIV	Cell Signaling Technologies	11967S	1:1000	WB
dsRNA	Millipore Sigma	MABE1134		IF
DRP1	Cell Signaling Technologies	5391S	1:1000	WB
FLAG	Cell Signaling Technologies	8146T	1:1000	WB

FLAG (M2)	Millipore Sigma	A8592	1:1000	IF
GAPDH	Protein Tech	6004-1	1:5000	WB
Galectin-3	Biolegend	125402	1:500	IF
GFP-HRP	Miltenyi Biotec	130-091-833	1:5000	WB
GFP	ThermoFisher Scientific	A10262	1:1000	IF
GM130	Cell signaling technologies	70767T	1:500	IF
LAMP1	Cell Signaling Technologies	9091s	1:500	IF
LAMP2	Santa Cruz	SC-18822	1:500	IF
LAMTOR4	Cell Signaling Technologies	13140S	1:200	IF
LBPA/BMP	Millipore Sigma	MABT837	1:500	IF
LC3	Santa Cruz	sc-271625	1:1000	WB
mCherry	ThermoFisher Scientific	M11217	1:1000/1:5,000	IF/WB
MIRO1	Millipore Sigma	HPA010687	1:1000	WB
MIRO2	Protein Tech	11237-1-AP	1:1000	WB
NPC1	Abcam	134113	1:1000/1:500	WB/IF
NPC2	Gift from P. Lobel		1:10000	WB
PDI	Cell Signaling Technologies	3501S	1:10,000	WB
PDH	Abcam	ab110333	1:500	IF
PLA2G15 (LYPLA3)	Santa Cruz	Sc-376078	1:500	WB
Rab7a	Cell Signaling Technologies	9367	1:200	IF
STX17	Abcam	ab316119	1:1000	WB
TGN46	BioRad	AHP500GT	1:500	IF
TOMM20	Santa Cruz	sc-17764	1:500	IF
TOMM20	Cell Signaling Technologies	42406	1:2000	WB
ULK1	Cell Signaling Technologies	8054S	1:1000	WB
V5	Protein Tech	14440-1-AP	1:500	IF
VPS11	Santa Cruz	sc-515094	1:1000	WB
VPS29	Cell Signaling Technologies	73540	1:1000	WB
VPS35	Abcam	Ab10099	1:1000/1;500	WB/IF
VPS39	Santa Cruz	SC-514762	1:500	WB
VPS41	Santa Cruz	SC-377118	1:500	WB

Table. S3 Other important reagents used in this study

**Reagent**	**Source**	**Product Number**	**Applications**
Bafilomycin	Milipore Sigma	88899-55-2	V-ATPase inhibitor

(S,S) LBPA/BMP	Echelon bioscience	L-B181	BMP addition
CellMask	Thermos fisher	H32722	Labels entire cells
Chloroquine	Milipore Sigma	50-63-5	V-ATPase inhibitor
Filipin	Milipore Sigma	F9765	Probes free cholesterol
LLOMe	Milipore Sigma	16689-14-8	Lysosome damage reagent
Monencin	Milipore Sigma	1445481	Increases lysosome pH
siMIRO1	Horizon discovery	L-010365-01-0005	RNAi
siMIRO2	Horizon discovery	L-008340-01-0005	RNAi
siDRP1	Horizon discovery	L-012092-00-0005	RNAi
siATG5	Horizon discovery	J-004374-07-0002	RNAi
siATG7	Horizon discovery	L-020112-00-0005	RNAi
siULK1	Horizon discovery	J-005049-05-0002	RNAi
U18666A	Milipore Sigma	U3633	NPC1 inhibitor
