# Supplementary material for: Manipulation of Host Cholesterol by SARS-CoV-2
Source: bioRxiv. 2024 Nov 14:2024.11.13.623299. Preprint. [Version 1] doi: 10.1101/2024.11.13.623299 (PMC11601339; doi:10.1101/2024.11.13.623299)
Supplement: Supplementary file 1 [file media-1.pdf]

SUPPLEMENTAL INFORMATION

Supplemental figure legends

Fig. S1. SARS-CoV-2 infection sequestrate cholesterol in lysosomes
A. A549-hACE2 cells were infected with SARS-CoV-2, and the infection rate was determined by immunostaining of dsRNA and quantified by high-content imaging system. **B.** An additional line of A549 cells stably expressing human ACE2 were infected, fixed at 24 h post-infection, stained with the antibodies against dsRNA and filipin, and imaged with confocal microscope. Note that the dsRNA-positive cell showed increased filipin intensity, compared to the dsRNA-negative cell. Scale bars, 5 µm.

Fig. S2. Confirmation of CRISPR-Cas9 gene knock out (KO) by immunoblotting.
A549-hACE2 cells were transfected with the indicated control or SARS-CoV-2 protein-encoded plasmids, fixed at 24 h post-transfection, stained with the antibodies against GFP or mCherry and filipin, and imaged with confocal microscope. Scale bars, 5 µm.

Fig. S3. Characterization of ORF3a-VPS39 interaction
A. A549-hACE2 cells were infected with SARS-CoV-2 and lysed at 24 h post-infection. Cell lysates were subjected to immunoblotting with using the indicated antibodies. **B-D.** HeLa cell were transfected with mCh-VPS39 construct alone (B) or co-transfected with mCh-VPS39 and ORF3a-GFP mutant constructs (C,D). The cells were fixed at 24 h post transfection, immunostained with the antibodies against GFP and mCherry, and imaged with confocal microscope. Note that mCh-VPS39 alone displayed cytosolic distribution (B, left panel), but when ORF3a was present, mCh-VPS39 formed puncta, colocalized with ORF3a (B, right panel). This distribution alteration was used as an indicator of ORF3a-VPS39 interaction in examining those ORF3a mutants (C,D). Scale bars, 5 µm.

Fig. S4. The impacts of ORF3a-VPS39 interaction to lysosome integrity
A,B. HeLa Flp-In cells were fixed, stained with BODIPY, and quantified for lipid droplets by high-content imaging. C. Wildtype HeLa cells were treated with the indicated drugs, stained with filipin, and quantified by high-content imaging. **D.** Example images of confocal microscopy, showing LLOMe treatment induced galectin-3 puncta (lysosome damage) and increased filipin puncta. **E.** HeLa Flp-In cells were examined for cathepsin L activity using a commercial kit. F. HeLa Flp-In cells were examined by immunofluorescence of galectin-3. No

puncta were observed by confocal microscopy. **G,H.** Cell lysates from HeLa Flp-In cells (G) or SARS-CoV-2-infected A549-hACE2 cells (24 h post-infection, H) were subjected to immunoblotting with the indicated antibodies. *p* values were determined using *t* test (B, CoV-2 vs W193A) or one-way ANOVA. *, $p < 0.05$, **, $p < 0.01$, ***, $p < 0.001$, **** or ####, $p < 0.0001$, n.s., no significant difference. Scale bars, 5 μm. Control (Ctrl), linker peptide, CoV, SARS-CoV ORF3a, CoV-2, SARS-CoV-2 ORF3a, W193A, SARS-CoV-2 ORF3a-W193A mutant.

Supplemental tables

Table. S1 cDNA constructs used in this study

Insert	Vector	Tag	Remarks
NPC2	mcherry-N1	c-mCherry	Gift from Dr. P. Lobel (Rutgers University)
SARS-CoV2-NSP1-strep	pLVX-EF1alpha-IRES-Puro	c-Strep	Addgene #141367
NSP2-strep	pLVX-EF1alpha-IRES-Puro	c-Strep	Addgene #141368
NSP3-GFP	pEGFP-N1	c- GFP	Addgene #165108
NSP4-mch	pmCherry-N1	c-mcherry	Addgene #165132
NSP5-strep	pLVX-EF1alpha-IRES-Puro	c-Strep	Addgene #141370
NSP6-mch	pmCherry-N1	c-mcherry	Addgene #165133
GFP- NSP7	pEGFP-C1	n-GFP	Addgene #165112
NSP8-GFP	pEGFP-N1	c-GFP	Addgene #165113
NSP9-strep	pLVX-EF1alpha-IRES-Puro	c-strep	Addgene #141375
NSP10-GFP	pEGFP-N1	c-GFP	Addgene #165115
NSP11-strep	pLVX-EF1alpha-IRES-Puro	c-Strep	Addgene #141377
GFP-NSP12	pEGFP-C1	n-GFP	Addgene #165117
NSP13-mch	pmCherry-N1	c-mcherry	Addgene #165136
NSP14-mch	pmCherry-N1	c-mcherry	Addgene #165137
NSP15-GFP	pEGFP-C1	n-GFP	Addgene #165120
ORF3a-CoV-2-GFP	pEGFP-N1	c-EGFP	This study
ORF3a-CoV-2-V5			This study
ORF3a-CoV-GFP	pEGFP-N1	c-GFP	Addgene # 165121
ORF3b-strep	pLVX-EF1alpha-IRES-Puro	n-strep	Addgene #141384
ORF6-strep	pLVX-EF1alpha-IRES-Puro	c-strep	Addgene #141387
ORF7a-strep	pLVX-EF1alpha-IRES-Puro	c-strep	Addgene #141388
ORF7b-strep	pLVX-EF1alpha-IRES-Puro	c-strep	Addgene #141389
ORF8-GFP	pmCellFree_KA1	n-GFP	Addgene 169395
GFP-ORF9b	pEGFP-C1	n-GFP	Addgene #165122

ORF10-strep	pLVX-EF1alpha-IRES-Puro	c-Strep	Addgene #141394
GFP-E	pEGFPC1	n-GPF	Addgne # 165123
GFP-M	pEGFPC1	n-GFP	Addgene #165124
N-strep	pLVX-EF1alpha-IRES-Puro	c-Strep	Addgene #141391
Spike	pGBW-m4137386	No tag	Addgene 149540
mCh-VPS39	pmCherry-C1	n-mcherry	Previous study
pDEST-Flp-In-linker-APEX2	pDEST-Flp-In_ORF_APEX2	c-FLAG-APEX2	This study
pDEST-Flp-In-ORF3a-CoV-APEX2	pDEST-Flp-In_ORF_APEX2	c-FLAG-APEX2	This study
pDEST-Flp-In-ORF3a-CoV-2-APEX2	pDEST-Flp-In_ORF_APEX2	c-FLAG-APEX2	Previous study
pDEST-Flp-In-ORF3a-CoV-2-W193A-APEX2	pDEST-Flp-In_ORF_APEX2	c-FLAG-APEX2	This study
GST-D4H-mcherry	pGEX-KG	n-GST, c-mCherry	Addgene #134604

Table. S2 Antibodies used in this study

**Antibody**	**Source**	**Product Number**	**Dilution**	**Applications**
dsRNA	Millipor Sigma	MABE1134		IF
Calnexin	Cell Signaling Technologies	2679s	1:8000	WB
Cathepsin D	Proteintech	219361	1:4000	WB
CD-MPR	Developmental Studies Hybridoma Bank	22d4	1:50	WB
CI-MPR	Abcam	124767	1:500	IF
CI-MPR	Proteintech	20253-1-AP	1:1000/1:500	WB/IF
Diaskedin (C10orf32)	Millipor-Sigma	HPA037648	1:1000	WB
EEA1	BD Biosciences	610457	1:200	IF
EEA1	Cell Signaling Technologies	3288	1:100	IF
Flag	Cell Signaling technologies	8146T	1:1000	WB
GAPDH	Proteintech	6004-1	1:5000	WB
GFP-HRP	Miltenyi Biotec	130-091-833	1:5000	WB
GFP	ThermoFisher Scientific	A10262	1:1000	IF
LAMP1	Cell Signaling Technologies	9091s	1:500	IF
LAMP2	Santa Cruz	SC-18822	1:500	IF
LAMTOR4	Cell Signaling Technologies	13140S	1:200	IF
mCherry	ThermoFisher Scientific	M11217	1:1000/1:5,000	IF/WB
NPC1	Abcam	134113	1:1000/1:500	WB/IF

NPC2	Gift from P. Lobel		1:10000	WB
PDI	Cell Signaling Technologies	3501S	1:10,000	WB
Rab5	Cell Signaling Technologies	3547	1:100	IF
Rab7a	Cell Signaling Technologies	9367	1:200	IF
RFP	ThermoFisher Scientific	R10367	1:1000	IF
TGN46	BioRad	AHP500GT	1:500	IF
VPS39	Santa Cruz	SC-514762	1:500	WB
VPS41	Santa Cruz	SC-377118	1:500	WB
V5	Protein Tech	14440-1-AP	1:500	IF
GM130	Cell signaling technologies	70767T	1:500	IF
LBPA/BMP	Millipore sigma	MABT837	1:500	IF
ACE2	Cell signaling technologies	4355	1:1000	WB
VPS11	Santa Cruz	sc-515094	1:1000	WB
FLAG (M2)	Milipore Sigma	A8592	1:1000	WB

Table. S3 Other important reagents used in this study

**Reagent**	**Source**	**Product Number**	**Applications**
Filipin	Milipore Sigma	F9765	Probes free cholesterol
CellMask	Thermos fisher	H32722	Labels entire cells
Bodipy	Thermo Fisher	D3861	Probes lipid droplets
U18666A	Milipore Sigma	U3633	NPC1 inhibitor
Monencin	Milipore Sigma	1445481	Increases lysosome pH
Bafilomycin	Milipore Sigma	88899-55-2	V-ATPase inhibitor
Chloroquine	Milipore Sigma	50-63-5	V-ATPase inhibitor
LLoMe	Milipore Sigma	16689-14-8	Lysosome damage reagent
Cathepsin L activity kit	Biorad	ICT941	Cathepsin L activity

Figure S1

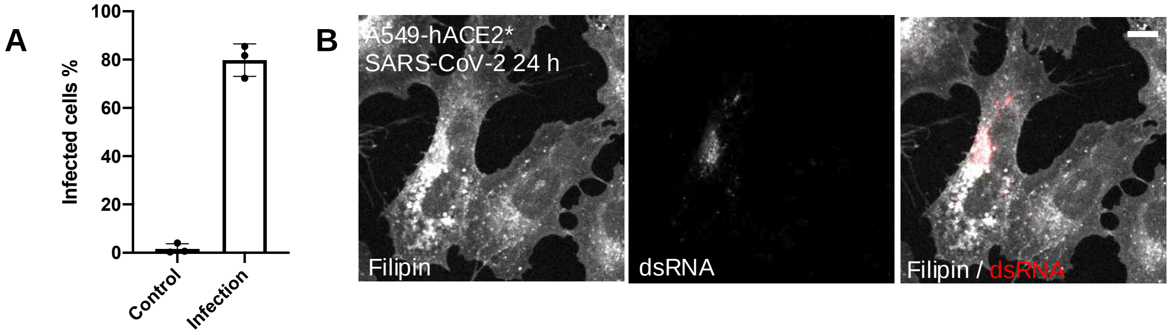
A
100
80
60
40
20
0
Infected cells %
Control
Infection
B
A549-hACE2*
SARS-CoV-2 24 h
Filipin
dsRNA
Filipin / dsRNA

Figure S2

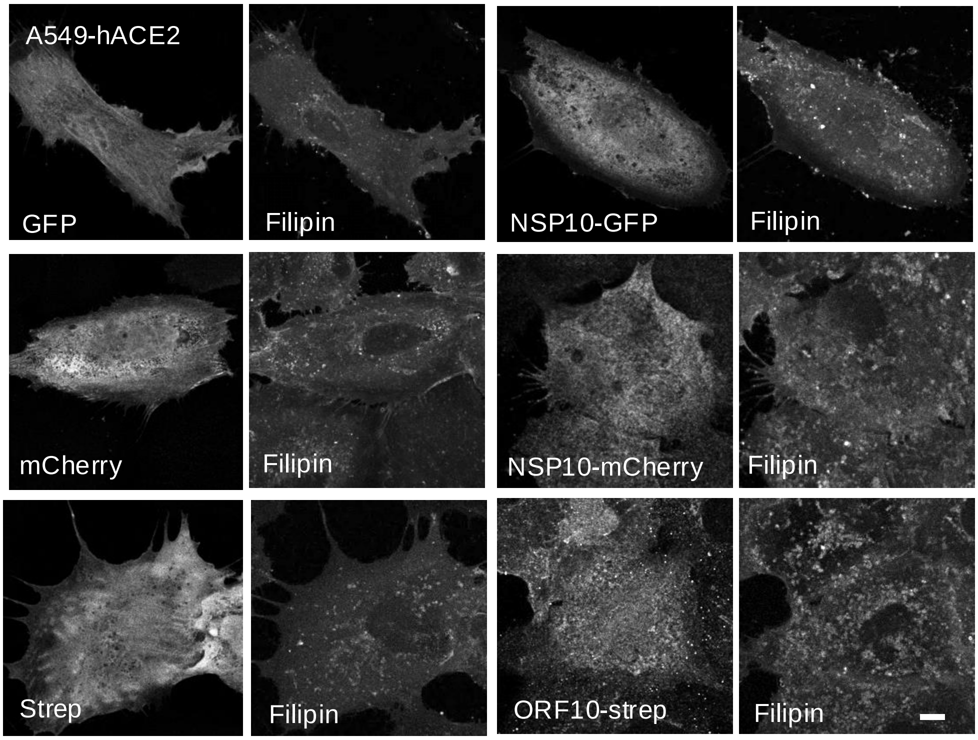
A549-hACE2
GFP
Filipin
NSP10-GFP
Filipin
mCherry
Filipin
NSP10-mCherry
Filipin
Strep
Filipin
ORF10-strep
Filipin

Figure S3

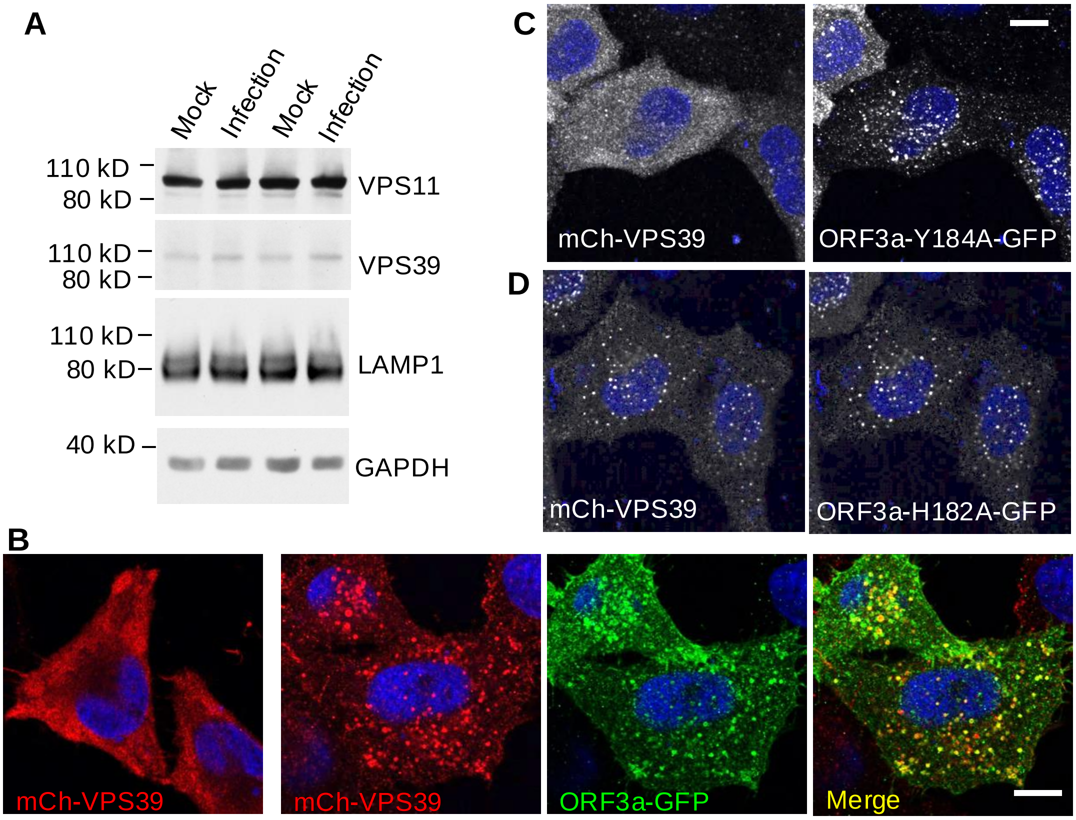
A
Mock
Infection
Mock
Infection
110 kD
80 kD
VPS11
110 kD
80 kD
VPS39
110 kD
80 kD
LAMP1
40 kD
GAPDH
B
mCh-VPS39
mCh-VPS39
ORF3a-GFP
Merge
C
mCh-VPS39
ORF3a-Y184A-GFP
D
mCh-VPS39
ORF3a-H182A-GFP

Figure S4

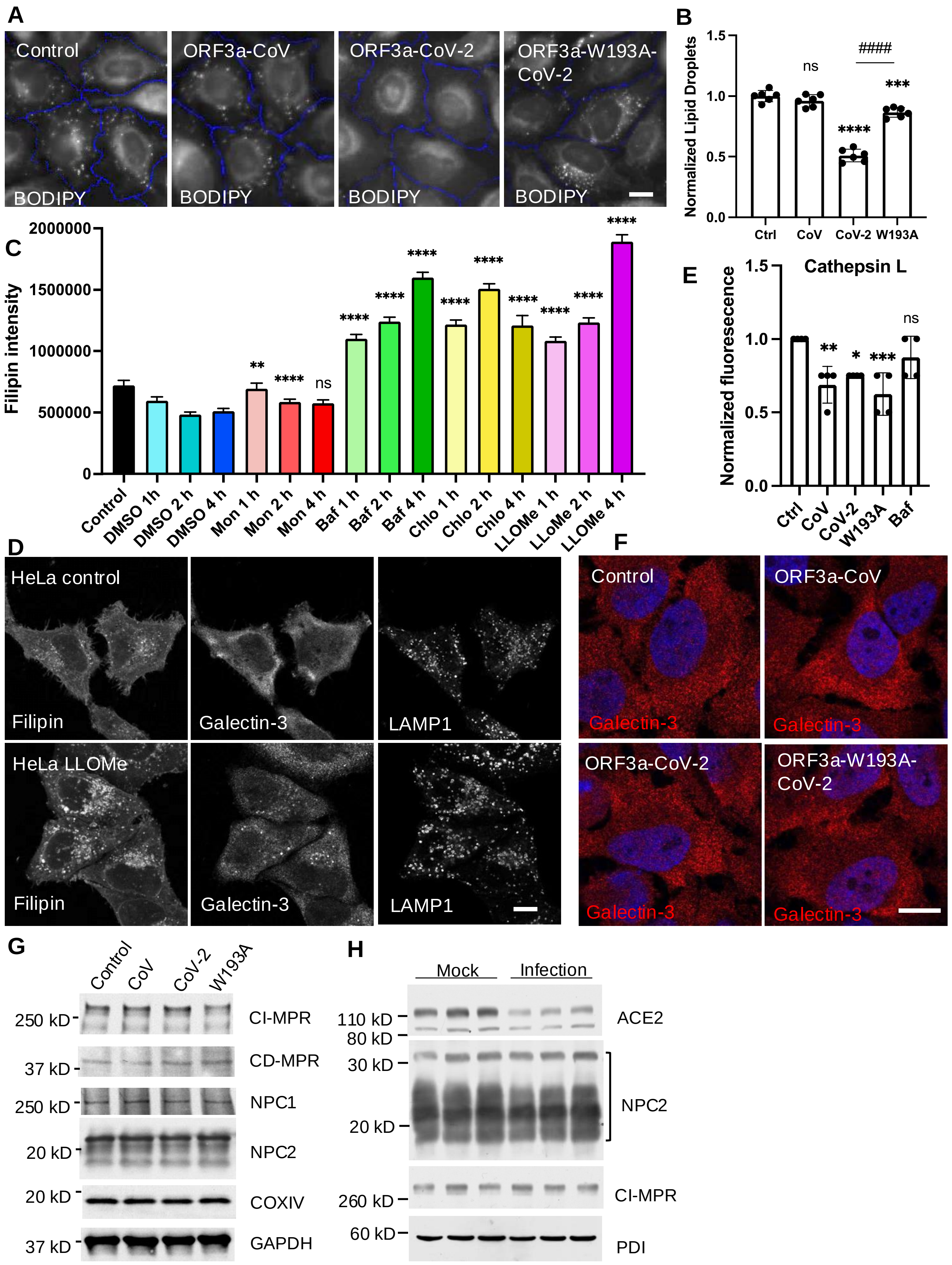
A
Control
ORF3a-CoV
ORF3a-CoV-2
ORF3a-W193A-CoV-2
BODIPY
B
Normalized Lipid Droplets
ns
####
***
****
Ctrl
CoV
CoV-2
W193A
C
Filipin intensity
Control
DMSO 1h
DMSO 2 h
DMSO 4 h
Mon 1 h
Mon 2 h
Mon 4 h
Baf 1 h
Baf 2 h
Baf 4 h
Chlo 1 h
Chlo 2 h
Chlo 4 h
LLOMe 1 h
LLOMe 2 h
LLOMe 4 h
E
Cathepsin L
Normalized fluorescence
Ctrl
CoV
CoV-2
W193A
Baf
D
HeLa control
HeLa LLOMe
Filipin
Galectin-3
LAMP1
F
Control
ORF3a-CoV
ORF3a-CoV-2
ORF3a-W193A-CoV-2
Galectin-3
G
Control
CoV
CoV-2
W193A
250 kD
37 kD
20 kD
CI-MPR
CD-MPR
NPC1
NPC2
COXIV
GAPDH
H
Mock
Infection
110 kD
80 kD
30 kD
20 kD
260 kD
60 kD
ACE2
NPC2
CI-MPR
PDI
